# A Comprehensive Analysis of METTL1 to Immunity and Stemness in Pan-Cancer

**DOI:** 10.3389/fimmu.2022.795240

**Published:** 2022-03-31

**Authors:** Zijie Gao, Jianye Xu, Zongpu Zhang, Yang Fan, Hao Xue, Xing Guo, Lin Deng, Shaobo Wang, Rongrong Zhao, Ping Zhang, Gang Li

**Affiliations:** ^1^ Department of Neurosurgery, Qilu Hospital, Cheeloo College of Medicine and Institute of Brain and Brain-Inspired Science, Shandong University, Jinan, China; ^2^ Shandong Key Laboratory of Brain Function Remodeling, Jinan, China

**Keywords:** pan-cancer, METTL1, immunity, stemness, immunotherapy

## Abstract

**Background:**

Previous studies have reported the effect of N^7^-methylguanosine (m^7^G) regulator methyltransferase like-1 protein (METTL1) in tumor initiation, metastasis, and chemosensitivity. However, the relationship between METTL1 and cancer immune infiltration is not validated and the prognostic significance of METTL1 in pan-cancer remains unclear.

**Methods:**

Clinical parameters, including gender, age, lifetime, stage, and treatment response were analyzed to evaluate the prognostic significance of METTL1. To evaluate protein level of METTL1, the METTL1 activity was generated by single sample gene set enrichment analysis. The one-class logistic regression algorithm was used to calculate the stemness indices based on transcriptomics and methylation data of pan-cancer and pluripotent stem cells. The relationship between METTL1 expression or activity and tumor immune infiltration were analyzed to explore the significance of METTL1 in tumor immunotherapy. Meanwhile, the correlation between three immunotherapeutic biomarkers and METTL1 was investigated. Finally, to calculate the association between drug sensitivity and METTL1 expression, spearman correlation analysis was performed.

**Results:**

METTL1 was not intimately related to gender, age, tumor stage, or treatment outcome of the various cancers, but it displayed potential prognostic significance for evaluating patient survival. High METTL1 expression was related to tumor progression-relevant pathways. Moreover, METTL1 exhibited a distinct correlation with tumor immune microenvironment infiltration and stemness indices. In the anti-PD-L1 cohort, patients in treatment response group exhibited significantly higher METTL1 expression than those in the no/limited response group. Further analysis showed that tumor cell lines with higher METTL1 expression were more sensitive to drugs targeting chromatin histone methylation, ERK-MAPK and WNT signaling pathways.

**Conclusion:**

This study provides insight into the correlation of METTL1 with tumor immune infiltration and stemness in pan-cancer, revealing the significance of METTL1 for cancer progression and guiding more effective and generalized therapy strategies.

## Introduction

Generation of the ‘‘epitranscriptome’’ through posttranscriptional RNA modification promotes regulatory complexity of RNA structure and function. In the past few years, extended studies in RNA biology have disclosed over 160 posttranscriptional RNA modifications (1). N^7^-methylguanosine (m^7^G), an electropositive modification at the RNA 5’ cap, is necessary for regulating mRNA export, translation, and splicing (2). Catalyzed by methyltransferase like-1 protein (METTL1), m^7^G tRNA modification is related to HeLa cell’s 5-fluorouracil sensitivity (3). In addition, METTL1-dependent m^7^G regulates miRNA structure and biogenesis (4). METTL1, localized on chromosome band 12q13, contains a conserved S-adenosylmethionine-binding motif and can be activated by loss of phosphorylation (5, 6). Normally, METTL1 is expressed in the kidney, thyroid, adrenal, appendix, and 23 other tissues (7). METTL1/WD repeat domain 4 (WDR4)-mediated m^7^G tRNA methylome modification is essential for ordinary RNA translation, regulating self-renewal as well as differentiation in embryonic stem cells (8). Recently, many studies have reported that METTL1 is extraordinarily expressed in a variety of cancers, including hepatocellular carcinoma, colon cancer, intrahepatic cholangiocarcinoma, lung cancer, breast cancer, glioblastoma, certain sarcomas, and acute myelogenous leukemia, which links to tumor initiation, metastasis, and chemosensitivity (9–13). In addition, m^7^G Arg-TCT-4-1 tRNA modification mediated by METTL1 leads to carcinogenic transformation, resulting in overexpression of CDK4, Hmga2, Ash2l, Setdb1, and Ube2t (14). Immunotherapy, also known as immune checkpoint blockade (ICB) therapy, has delivered promising clinical outcomes in multifarious cancers. Nevertheless, it presents a limited response rate due to the complexity of tumor immune microenvironment and the impact of immune escape (15, 16). In this way, to improve the effectiveness of immunotherapy for pan-cancer, it is significant to investigate the immunosuppressive tumor microenvironment (TME) thoroughly. Recent studies have uncovered that posttranscriptional RNA modification takes a significant part in the formation of the tumor immune microenvironment (17–19).

Stemness, termed as the ability of self-renewal and differentiation from initial cells. The more primitive of tumor, the higher of stemness, and the more likely it is to have distant metastasis and multitherapy resistance, finally leading to cancer progression and poor prognosis (20–22). Tathiane M et al. obtained epigenetic and transcriptomic signatures from pluripotent stem cells as well as their differentiated progeny to generate stemness indices based one-class logistic regression (OCLR) machine learning algorithm (23). These stemness indices could be used to evaluate the degree of tumor dedifferentiation and survival prognosis of patients.

In our current study, the METTL1 expression pattern of pan-cancer was revealed and the fundamental effects of METTL1 on the immunosuppressive TME were examined. A variety of immunomodulators and immunotherapeutic biomarkers were investigated in this study. Moreover, the association between METTL1 and ICB therapy was investigated. Collectively, this study provides evidence for elucidating immunotherapeutic effect of METTL1 in a variety of cancers, which is likely to be instrumental for further research.

## Materials and Methods

### Data Acquisition

We obtained The Cancer Genome Atlas (TCGA, https://portal.gdc.cancer.gov) data: 33 types cancer RNA transcriptome data (Fragments Per Kilobase Million value), somatic mutation data, and clinical characteristics from University of California Santa Cruz (UCSC) Xena dataset portal (http://xena.ucsc.edu/) (24). Three immunotherapy research were finally enrolled in this study. IMvigor210 cohort with the RNA sequence information as well as clinical data were acquired from http://research-pub.gene.com/IMvigor210CoreBiologies/packageVersions/. The RNA sequencing data and clinical information of nivolumab-treated renal cell carcinoma (GSE 67501) cohort and pembrolizumab-treated metastatic melanoma (GSE 78220) cohort were acquired from Gene expression omnibus database (GEO) respectively. Transcriptome data of cancer cells were obtained from the Cancer Cell Line Encyclopedia (CCLE; http://sites.broadinstitute.org/ccle/datasets) database and the drug sensitivity values of paired cell lines were acquired from Genomics of Drug Sensitivity in Cancer (GDSC; https://www.cancerrxgene.org/celllines) database.

### METTL1 Expression Among Clinical Traits in Pan-Cancer

We analyzed the expression level between tumor and normal groups in pan-cancer utilizing the “limma” package of R studio software. Besides, we grouped patients according to other clinical traits (age, gender, stage, status, and treatment outcome) to analyze the expression of METTL1. Furthermore, we investigated the prognostic value of METTL1 in pan-cancer utilizing univariate Cox regression analysis. Overall survival (OS), progression-free-survival (PFS), disease-free-survival (DFS), and disease-specific-survival (DSS) were selected to study the relevance between METTL1 expression and prognosis.

### Generation of METTL1 Activity

To examine the expression of METTL1 more comprehensive, we constructed METTL1 gene signature based on 100 genes which were most related to METTL1 expression in 33 cancers. Then the METTL1 activity was calculated by single sample gene set enrichment analysis (ssGSEA). Next, METTL1 activity was compared between tumor and normal tissue groups. Correlation between METTL1 activity and other clinical information was also investigated.

### Cell Lines and Cell Culture

Human glioma cell lines A172, U251, U118MG (Chinese Academy of Sciences Cell Bank), and LN229 (ATCC Cell Bank) were cultured in DMEM (Sigma, USA) supplemented with 10% FBS (Thermo Fisher Scientific, USA). Human nasopharyngeal carcinoma cell line CNE2 (ATCC Cell Bank) and human monocyte cell line THP-1 (Chinese Academy of Sciences Cell Bank) were cultured in RPMI-1640 (Sigma) supplemented with 10% FBS. THP-1 cells were incubated with 100 ng/ml phorbol 12-myristate 13-acetate Sigma) for 24 h to induce their differentiation into macrophages. The cell lines were incubated at 37℃ with 5% CO_2_.

### Small Interfering RNA (siRNA) and Plasmid Transfection

SiRNAs targeting METTL1 (GenePharma, China) and plasmid overexpression of METTL1 (GenePharma, China) were synthesized. SiRNAs and plasmids were transfected with Lipofectamine™ 3000 reagent (Thermo Fisher Scientific) according to the manufacturer’s protocol.

### Western Blotting

Proteins from GBM cells were obtained using RIPA lysis buffer (ThermoFisher, USA) containing a 1% protease and phosphate inhibitor cocktail. PVDF membranes (Bio-Rad, CA, USA) were incubated with specific antibodies after Western blotting was performed. The primary antibodies used in this study are as follows: METTL1 (Proteintech, USA) and β-actin (Cell Signaling Technology, USA).

### 5-Ethynyl-2′-Deoxyuridine (EdU) Cell Proliferation Assay

An EdU cell proliferation assay kit (RiboBio, #C10310-1; China) was used to evaluate the proliferative activity of GBM cells. The cell proliferation rate was assessed *via* the ratio of EdU-positive (red) cells to total Hoechst-positive (blue) cells.

### Transwell Assay

To evaluate migratory ability, U251 cells were added to the top chamber in DMEM without FBS, and the bottom chamber was filled with 10% FBS DMEM. To measure the ability to recruit macrophages, macrophages were added to the top chamber in RPMI-1640 without FBS, and the bottom chamber was filled with 10% FBS RPMI-1640 and growth media of different groups of CNE2 cells. After 24 h of incubation, the membrane was fixed in 4% paraformaldehyde and stained with crystal violet for 20 min and observed by microscopy.

### Relationship Between METTL1 Expression or Activity and Immunity

Estimation of Stromal and Immune Cells in Malignant Tumor Tissues Using Expression Data (ESTIMATE) algorithm was used to infer the degree of infiltration of stroma or immune cells into tumour based on RNA sequence profiles (25). Using ESTIMATE package, we calculated the stromal and immune score for each sample. Then we investigated the relationship between METTL1 expression or activity and four scores (Stromal score, Immune score, ESTIMATE score, and Tumor purity) generated by ESTIMATE. As a general deconvolution algorithm, CIBERSORT could assess the relative abundance of various tumor infiltrating immune cell subpopulations (26). The relationship between METTL1 expression or activity and 22 immune cell infiltration was calculated in 33 cancers respectively. In addition, using the TISIDB website (http://cis.hku.hk/TISIDB/index.php), we explored the relationship between METTL1 expression and three immunological modulators including immune stimulators, immune inhibitors, and major histocompatibility complex (MHC) molecules.

### Stemness Index Generated From OCLR

To quantify the tumor stemness, we trained a predictive model using OCLR based on mRNA expression or DNA methylation data obtained from PCBC dataset (https://progenitorcells.org/). Stemness index ranging from 0 to 1 is used to measure the resemblance among tumor cells and stem cells. OCLR-based models are trained to generate transcriptomic and epigenetic signatures. Transcriptomic and epigenetic signatures were then used to quantify the stem cell indices of TCGA samples. We used transcriptomic signatures to score TCGA cohort using spearman correlation for RNA expression data, whereas, for DNA methylation data, the linear model was applied to calculate the stemness indices. We could be able to reveal the stemness of GBM patients from gene expression and epigenetic features respectively.

### Relationship Between METTL1 Expression or Activity and Stemness

To reflect the relationship between METTL1 expression or activity and the self-renewal potential of tumor cells, we performed Spearman correlation analysis and visualised the results as heatmap.

### The Biological Significance of METTL1 Expression

To analyze the biological process of METTL1 in 33 cancers, we used “Gene set variation analysis (GSVA)” R-package to generate gene function enrichment analysis (27). “c2.cp. kegg.v7.4” as well as “c5. go.bp.v7.4” were received from Molecular Signatures Database (MSigDB)-v7.4 (http://www.gsea-msigdb.org/gsea/) for GSEA analysis.

### Cell Counting Kit -8 and Drug Dose-Response Curves

The CCK-8 was used to evaluate the cell viability. U118MG or U251 cells (5 × 10^3^ cells/well) were incubated in 96-well plates. Cells were treated with diverse drug concentration gradients (10, 20, 40, 80 160, and 320μM) for 24 hours. Then the CCK-8 solution (10 μL) was added to each well and the plates were incubated for 1 h at 37°C, and then the absorbance at 450 nm wavelength (OD450) was measured in a Perkinelmer EnSight. Data were normalized to max/min and the IC50 was visualized and calculated in Graphpad PRISM.

### Association Analysis Between Drug Sensitivity and METTL1 Expression

The expression of METTL1 in various cell lines were obtained from CCLE. The AUC value which indicated cellular sensitivity to drugs were obtained from GDSC. Then we performed spearman correlation analysis to estimate the association between drug sensitivity and METTL1 expression. A positive correlation indicates that cell lines with high METTL1 expression have higher AUC values and lower sensitivity to specific drugs, whereas a negative correlation indicates that cell lines with high METTL1 expression have lower AUC values and higher sensitivity to specific drugs. Besides, EPZ5676 and Ulixertinib were purchased from MedChemExpress (USA).

### Statistical Analysis

Using Spearman and Pearson coefficients, relationships between variables were analyzed. Two-group comparisons were performed with Student’s t-Test and represented as mean ± SD unless noted otherwise. Wilcoxon and one-way ANOVA test were applied for non-parametric data and parametric for comparisons among three or more groups, respectively. For survival analyses, applying the Kaplan-Meier approach and log-rank test, we acquired survival curves while assessed the statistical significance. All statistical analyses were two-sided and all statistical data analyses were carried out *via* Rstudio software. P < 0.05 was regarded as statistically significant.

## Results

### Clinical Landscape of METTL1 Expression in Pan-Cancer

**Figure 1:**
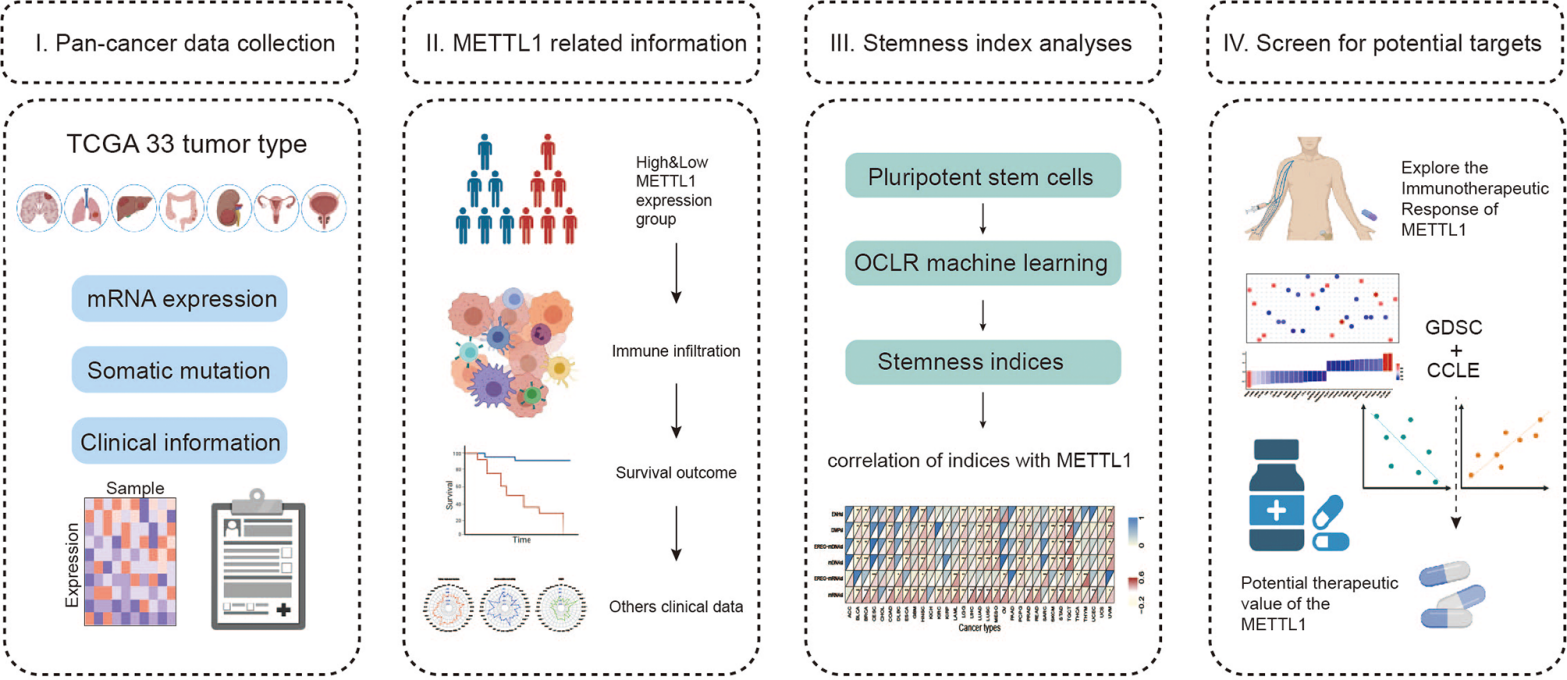
Landscape of this study workflow. I. The mRNA expression data, somatic information and clinical traits were collected for follow-up analysis. II. Patients were divided into high and low expression groups according to METTL1 expression, and then we compared the immune infiltration, tumor mutation burden, survival prognosis, etc. between the different groups. III. Stemness indices were generated using OCLR algorithm and correlation analysis between stemness indices and METTL1 expression was performed. IV. Exploring the immunotherapeutic response of METTL1 in diverse ICB cohorts and investigating the potential targeting therapeutic value of METTL1.

**Figure 2:**
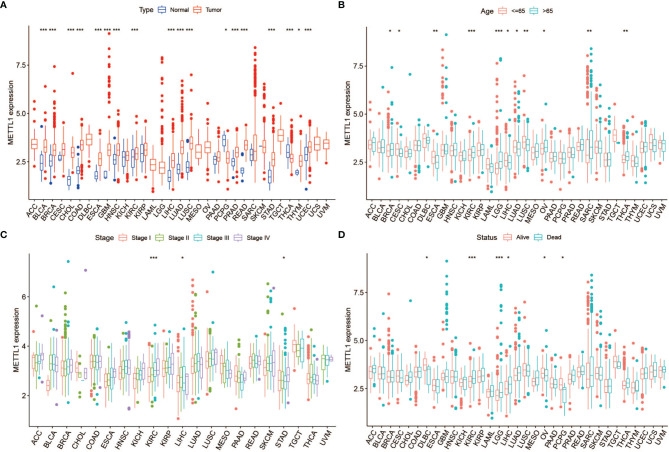
Correlation between METTL1 expression and clinical traits. **(A)** The expression of METTL1 between normal tissues and tumor among pan-cancer. **(B)** The relationship between METTL1 expression and age distribution. **(C)** The relationship between METTL1 expression and tumor stage. **(D)** The relationship between METTL1 expression and status. * means P < 0.05; ** means P < 0.01; *** means P < 0.001.

**Figure 1** shows the study details for a comprehensive prospect. **Table S1** presents the full names and abbreviations of the 33 cancers enrolled in this study. To identify the differences of METTL1 expression between tumours and normal tissues, we obtained METTL1 expression. As indicated in **Figure 2A** and **Table S2**, METTL1 was differentially expressed in 18 of 33 kinds of cancers (UCEC, THCA, THYM, ESCA, STAD, PCPG, PRAD, LIHC, LUAD, LUSC, KIRC, HNSC, READ, GBM, CHOL, COAD, BLCA, and BRCA). The expression of METTL1 was differentially higher in UCEC, THYM, STAD, READ, PRAD, LIHC, LUAD, LUSC, KIRC, HNSC, GBM, ESCA, CHOL, COAD, BLCA, and BRCA than in the corresponding normal tissues. In contrast, METTL1 expression was lower in PCPG and THCA. METTL1 was significantly highly expressed in elder patients of BRCA, KIRC, LGG, OV, SARC, and THCA cases, nevertheless the expression of METTL1 was weakly expressed in CESC, ESCA, LIHC, LUAD, as well as LUSC groups (**Figure 2B**). In addition, METTL1 expression was significantly associated with tumour stage in some cancers, such as KIRC, LIHC and STAD. (**Figure 2C**). In the meantime, the results illustrated significant differences based on gender in METTL1 expression of BRCA, KIBP, as well as SARC (**Figure S1A**). What is more, patients who were alive at the last follow-up had different METTL1 expression in DLBC, KIRC, LGG, LIHC, OV, and PCPG (**Figure 2D**). The patients’ treatment outcomes were associated with the METTL1 expression in KIBP and LGG (**Figure S1B**).

**Figure 3:**
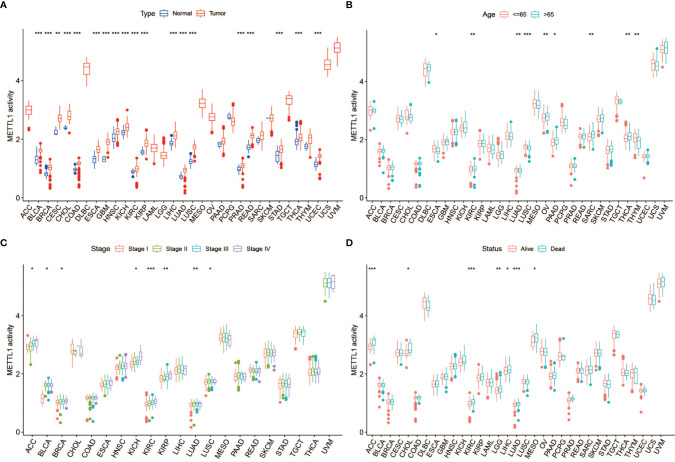
Correlation between METTL1 activity and clinical traits. **(A)** The differential activity of METTL1 between normal and tumor tissues among pan-cancer. **(B)** The relationship between METTL1 activity and age. **(C)** The relationship between METTL1 activity and tumor stage. **(D)** The relationship between METTL1 activity and status. * means P < 0.05; ** means P < 0.01; *** means P < 0.001.

To further assess the significance of the protein level of METTL1, we constructed METTL1 gene signature based the 100 genes which were most related to METTL1 expression in pan-cancer. METTL1 activity was generated using ssGSEA. As the results showed, METTL1 activity was significantly elevated in tumor group of BLCA, and UCEC, READ, THCA, STAD, PRAD, LUSC, LUAD, LIHC, ESCA, KIBP, KICH, KIRC, HNSC, GBM, CESC, CHOL, COAD as well as BRCA (**Figure 3A**; **Table S3**). In addition, the association between METTL1 activity and age, tumor stage, gender, survival, and treatment outcome were presented in **Figures 3B-D**, **S1C-D**. The results of **Figure S1E** demonstrated that six kinds of cancers (TGCT, DLBC, UVM, ACC, UCS, and SKCM) indicate similarly higher expression and activity of METTL1.

### Prognostic Prediction Function of METTL1 in Pan-Cancer

**Figure 4:**
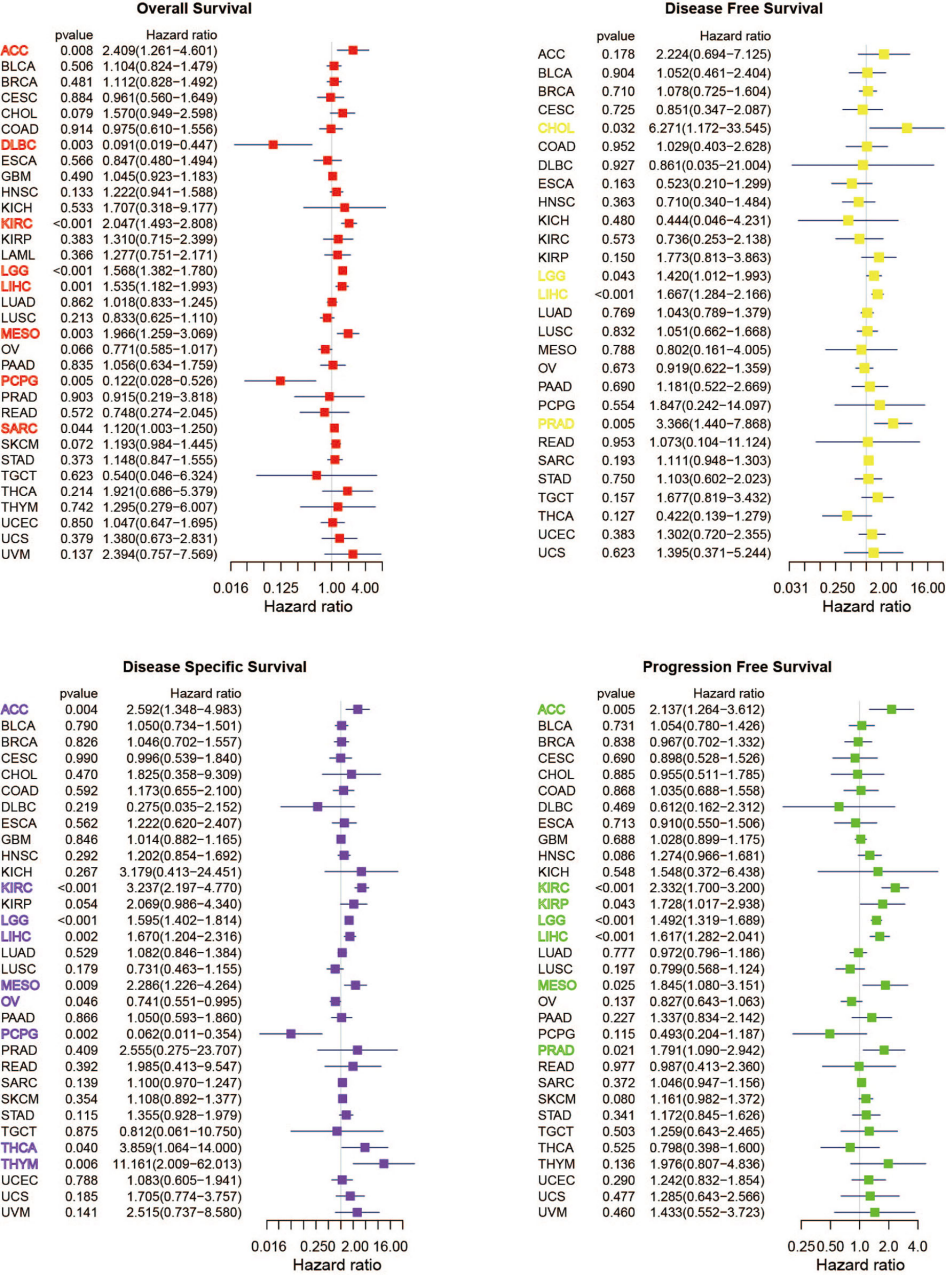
Univariate Cox regression analyses for METTL1 expression in pan-cancer. Forest plot visualizing the association of METTL1 expression and OS, DFS, DSS as well as PFS respectively among pan-cancer. Hazard ratio (HR) value > 1 represents risk factor, whereas HR value < 1 represents favorable factor.

A positive relationship was obvious between OS and METTL1 expression in ACC, MESO, KIRC, LGG, LIHC, and SARC, yet a negative correlation was identified in DLBC and PCPG as shown in the forest plots (**Figure 4**; **Table S4**). Regarding METTL1 expression and DFS, a significant positive association was observed in CHOL, LGG, LIHC, and PRAD (**Figure 4**; **Table S4**). In view of DSS, METTL1 expression had a favorable influence in OV and PCPG, however, it deemed to be a hazard factor in ACC, KIRC, LGG, LIHC, MESO, THCA, as well as THYM (**Figure 4**; **Table S4**). What’s worse, METTL1 expression was presented as a hazard factor among ACC, KIRC, KIRP, LGG, LIHC, MESO, and PRAD in the PFS forest plot (**Figure 4**; **Table S4**). **Figure S2** showed significant correlation between METTL1 expression and OS, DFS, DSS, as well as PFS in some types of cancer. To further evaluate the prognosis value of METTL1 among pan-cancer, we performed receiver operating characteristic curve (ROC) analysis and exhibited results in **Figure S3**. As the results exhibited, METTL1 expression exhibited powerful capacity of OS prediction in ACC, DLBC, LGG, MESO, and PCPG patients accurately, with an average AUC about 0.7 during a follow-up of 3 years. For DFS at two years later, METTL1 expression showed strong predictive power in CHOL, with an AUC above 0.8. For DSS, the expression of METTL1 presented powerful prediction capacity in ACC, LGG, MESO, and PCPG, with an average AUC about 0.7. However, the predictive power of METTL1 expression for PFS was not satisfactory in ACC, KIRC, KIRP LIHC, LGG, and PRAD, with the AUC less than 0.7 during a follow-up of 3 years. In addition, the expression of METTL1 was an independent risk factor for LGG and LIHC (**Figures S5C, D**).

We performed survival analysis to further assess the prognostic significance of METTL1 activity. Interestingly, a positive relationship was obvious between OS and METTL1 activity in ACC, KIRC, KIRP, LGG, LIHC, LUAD, MESO, SARC and SKCM, yet a negative correlation was identified in OV as shown in the forest plots (**Figure S4**). With regard to METTL1 activity and DFS, a significant positive association was observed in LIHC (**Figure S4**). In term of DSS, METTL1 activity deemed to be a hazard factor in ACC, KICH, KIRC, KIRP, LGG, LIHC, LUAD, MESO, as well as SKCM (**Figure S4**). In addition, METTL1 activity was presented as a hazard factor among ACC, KIRC, KIRP, LGG, LIHC, MESO, PRAD, and SKCM in the PFS forest plot (**Figure S4**).

### Influence of METTL1 on Tumor Progression

**Figure 5:**
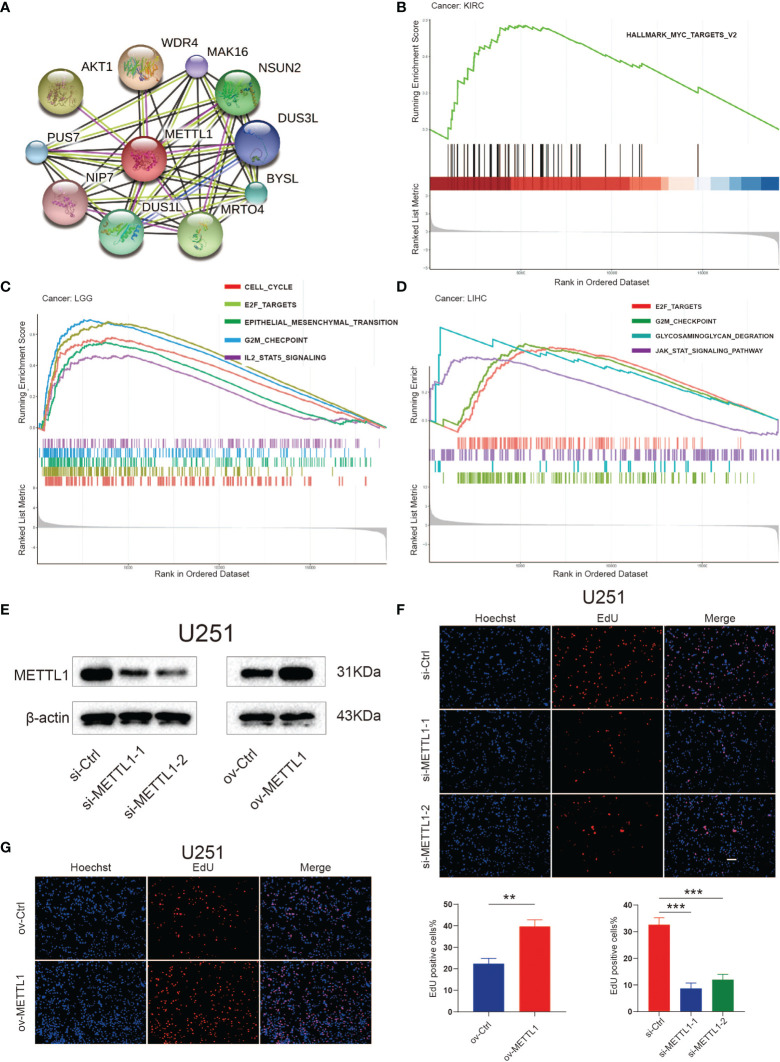
Functional analyses of METTL1. **(A)** PPI network visualized the potential proteins that interact with METTL1 and play a biological regulating function of METTL1. Line colors indicates the prediction methods; “green” indicates neighborhood, “red” indicates gene fusion, “blue” indicates co-occurrence, “black” indicates co-expression, “pink” indicates experiments, “wathet” indicates databases, “grass green” indicates textmining. **(B-D)** GSEA results based on Hallmark and KEGG dataset among KIRC, LGG, and LIHC. **(E)** Western blot analysis was performed to assess the expression levels of METTL1. β-actin was used as a loading control. **(F, G)** EdU was performed in U251 transfected si-control, si-METTL1#1, si-METTL1#2, vector and ov- METTL1 cells (scale bar = 100 μm). ** means P < 0.01; *** means P < 0.001.

To suppose the web of possible correlation for METTL1 and top ten candidate proteins, the protein-protein interaction (PPI) analysis was performed. We found that there were interactions between METTL1 and WDR4 (score = 0.979), AKT serine/threonine kinase 1 (AKT1, score = 0.918), mRNA turnover 4 homolog (MRTO4, score = 0.849), NOP2/Sun domain family member 2 (NSUN2, score = 0.815), dihydrouridine synthase 1-like (DUS1L, score = 0.801), bystin-like (BYSL, score = 0.794), pseudouridylate synthase 7 homolog (PUS7, score = 0.794), dihydrouridine synthase 3-like (DUS3L, score = 0.788), MAK16 (score = 0.784),nuclear import 7 homolog (NIP7, score = 0.780) (**Figure 5A**). All these proteins are critical in posttranscriptional RNA modification processing.

Considering the robust correlation between METTL1 and KIRC, LGG, and LIHC, we enquired the potential pathways concerning METTL1 signaling in pan-cancer using GSEA. The **Figures 5B-D** manifest that genesets from proliferation and metastasis related pathways, containing the E2F, JAK-STAT, and epithelial-mesenchymal transition signaling pathways, prefer being enriched in the high METTL1 cases of KIRC, LGG, as well as LIHC. The enrichment result for other tumor types were presented in **Figure S9**.

To assess the role of METTL1 in diverse cellular processes, we knocked down and overexpressed METTL1 in U251, respectively, and performed several experiments. Downregulation of METTL1 resulted in a significant decrease in the EdU-positive cell percentage, whereas overexpression of METTL1 increased EdU-positive cell percentage (**Figures 5E–G**). Transwell assays revealed that METTL1 silencing reduced the number of cells migrating to the membrane and METTL1 overexpression increase it (**Figure S5A**).

### Association Between Immune-Related Factors and METTL1

**Figure 6:**
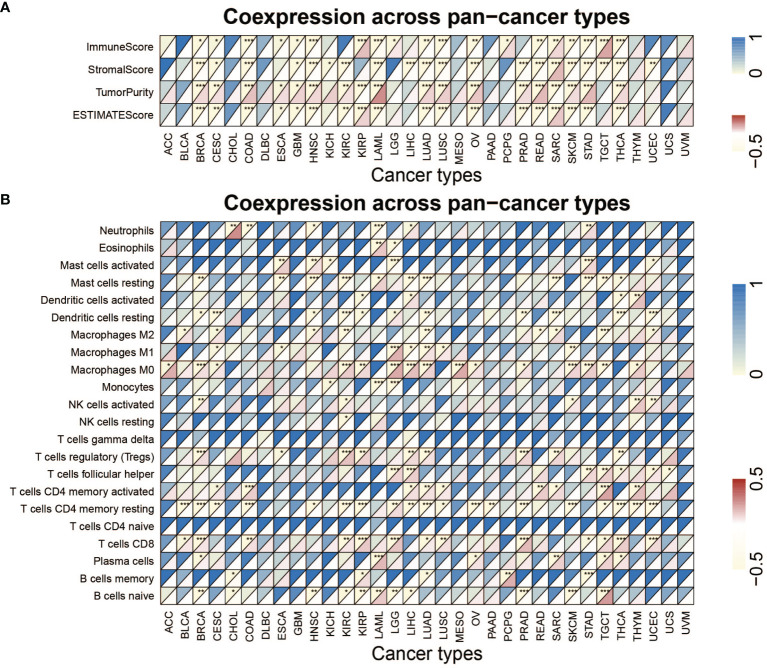
Analysis of METTL1 expression related immune characteristics. **(A)** Heatmap visualized the relationship between METTL1 expression and immune score, stromal score, tumor purity as well as ESTIMATE score in pan-cancer. **(B)** Heatmap showed correlation between METTL1 expression and 22 immune cell infiltration value obtained by CIBERSORT algorithm among pan-cancer. Upper half of each grid exhibited the p value and lower half exhibited the correlation coefficient. * means P < 0.05; ** means P < 0.01; *** means P < 0.001.

**Figure 6A** and **Table S5** list the stromal score, immune score, ESTIMATE score, as well as tumor purity. Notably, METTL1 expression was positively associated with the SARC stromal score, whereas it was positively related to the KIRP, LGG, PCPG, SARC, and TGCT immune score. In HNSC, METTL1 expression was positively associated with the stromal score, immune score, as well as ESTIMATE score. In addition, M2 macrophage infiltration was positively associated with METTL1 expression in HNSC (**Figure 6B**; **Table S6**). Interestingly, we found that METTL1 knockdown significantly inhibited the capability of conditioned medium from CNE2 cells to recruit THP1-differentiated macrophage and METTL1 overexpression promoted it (**Figure S5B**). METTL1 expression was positively associated with regulatory T cell content in BRCA, KIRC, KIRP, LIHC, LUAD, PRAD, and SARC for immune cell infiltration (**Figure 6B**; **Table S6**). In TGCT, METTL1 expression was negatively associated with resting mast cell, M2 macrophage, and M0 macrophage infiltration but positively correlated with activated memory CD4+ T cells, follicular helper T cells, CD8 T cell, and naive B cell infiltration (**Figure 6B**; **Table S6**). In addition, Figures S6A, B showed the association between METTL1 activity and immune infiltration. What’s more, the correlation between immune modulators and METTL1 expression was analyzed. As depicted in **Figure S7A**, the correlation between METTL1 expression and twenty-four immune inhibitors were studied. METTL1 expression was positively correlated with PVRL2 in nearly all cancers, whereas, it was negatively associated with KDR and CD274 in nearly all cancers. In addition, the analyses of forty-five immune stimulators showed that METTL1 expression was positively related to TNFRSF18 as well as negatively associated with TNFSF15 and IL6R in nearly all cancers (**Figure S7B**). **Figure S7C** emerges that METTL1 expression was positively correlated to TAPBP and HLA-A in nearly all types of cancers. In the contrary, we uncovered a negative association between METTL1 expression and HLA-DOA as well as HLA-E among nearly all cancers.

**Figure 7:**
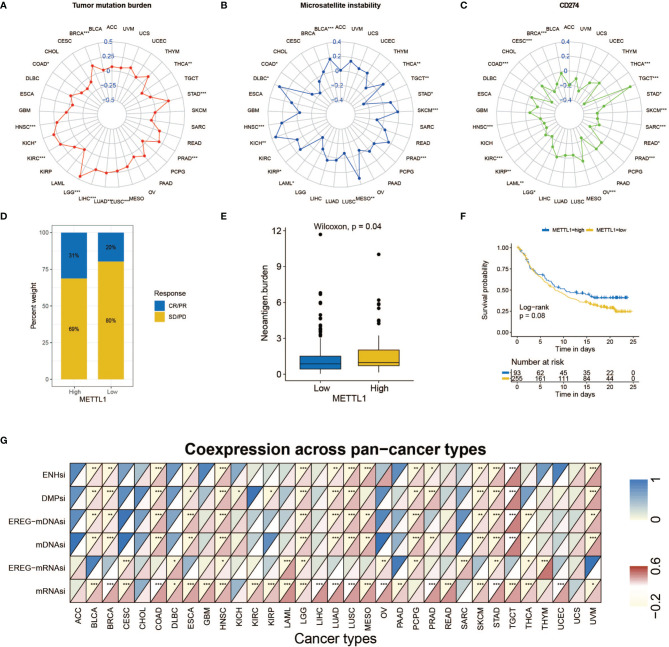
Role of METTL1 expression in response to ICB therapy. **(A-C)** Radar chart visualized the relationship between METTL1 expression and TMB **(A)**, MSI **(B)** and CD274 expression value respectively **(C)**. The dots in the radar chart indicates the correlation coefficient. **(D)** The column chart showed the proportion of patients with low or high METTL1 expression who responded to PD-L1 block immunotherapy. **(E)** Differences in neoantigen burden between low and high METTL1 expression groups (P = 0.04, Wilcoxon test). **(F)** Kaplan-Meier curve showed the survival of the high and low METTL1 expression patient groups in anti-PD-L1 immunotherapy cohort (IMvigor210 dataset; P = 0.08, Log-rank test). **(G)** Heatmap exhibited the relationship between METTL1 expression and stemness indices among pan-cancer. The upper half of each grid exhibited the p value and the lower half exhibited the correlation coefficient. * means P < 0.05; ** means P <0.01; *** means P < 0.001.

**Figure 7A** explained that METTL1 expression was positively correlated to the tumor mutational burden (TMB) in STAD, PRAD, LUAD, LUSC, LIHC, LGG, KICH, KIRC HNSC, and BRCA, while a negative association was found in COAD and THCA. For microsatellite instability (MSI), a positive association in BLCA, DLBC, HNSC, KICH, KIBP, MESO, PRAD, SKCM, STAD, and THCA, as well as a negative association in COAD, LAML, and TGCT was identified (**Figure 7B**). In addition, **Figure 7C** indicates METTL1 expression is negatively related to the CD274 in THCA, SARC, STAD, SKCM, PRAD, OV, LGG, LAML, KIRC, KIBP, HNSC, COAD, CESC, and BRCA, whereas positively in TGCA.

### Correlation Between METTL1 Expression and Response to ICB Therapy With PD-1/L1 Blockers

Significant efforts have been performed to distinguish biomarkers to foretell the immunotherapy response; some previously studied effective biomarkers include the TMB and the PD-1/L1 protein expression level (28). Considering that the METTL1 expression appears to be associated with the TME, we probed the value of the METTL1 on predicting the response to ICB therapy. Three immunotherapy cohorts, two anti-PD-1 cohorts (GSE 67501; GSE 78220) and an anti-PD-L1 cohort (IMvigor210) were involved (29–31). The patients in the IMvigor210 cohort demonstrated various degrees of response to anti-PD-L1 blockers, consisting of stable disease (SD), progressive disease (PD), partial response (PR), as well as complete response (CR). We discovered that METTL1 expression in response group was significantly higher than those in no/limited response groups (**Figure 7D**). The METTL1 expression in excluded immune phenotype group was obviously lower than that in disease group (**Figure S8A**). Tumor neoantigen burden reflects the response to ICB therapy directly and has been deemed as a favorable prognostic factor among patients. We found that patients with high METTL1 expression had high neoantigen burden (**Figure 7E**). However, the association between METTL1 expression and survival was not obvious (**Figure 7F**). For patients without platinum therapy, METTL1 expression in response group was significantly higher than those in no/limited response groups and high-METTL1-expression cases had favorable prognosis (**Figures S8B, C**). No evident difference of METTL1 expression was detected between the non-response and response groups in two anti-PD-1 cohorts (**Figures S8D, E** **)**. We found that 30 cancers, except for ACC, CHOL, and UCS, revealed correlation between the stemness indices and METTL1 expression (**Figure 7G**; **Table S7**). In addition, **Figure S6C** showed the association between METTL1 activity and stemness indices.

### Potential Therapeutic Value of METTL1 Expression

**Figure 8:**
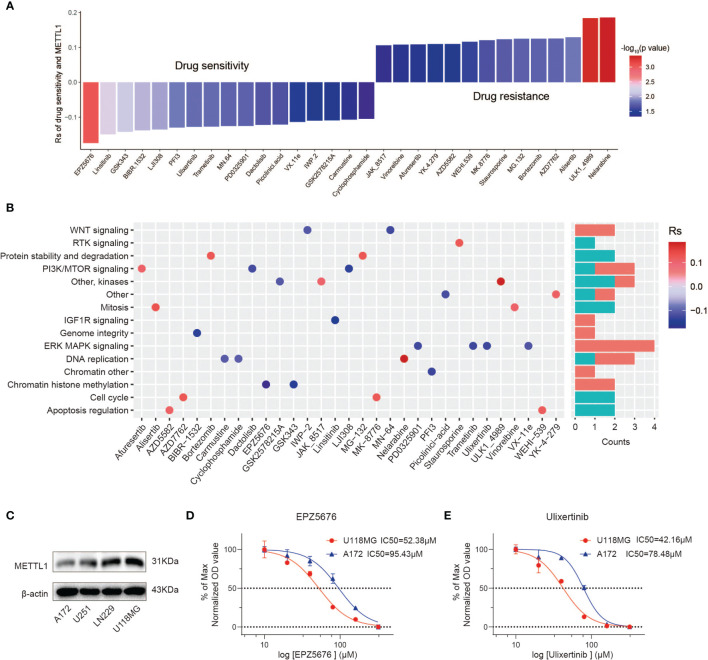
The relationship between METTL1 expression and response to drug sensitivity. **(A)** The relationship between METTL1 expression and drug sensitivity calculated by Spearman algorithm. The color of each column represents the p value, whereas the height of each column represents the correlation coefficient. Rs represents the drug sensitivity correlated with the METTL1 expression. **(B)** Dot plot visualized the signal pathways targeted by drugs which were sensitivity (blue) or resistant (red) to the METTL1 expression. The bar plot on the right represented drugs’ number of each targeted pathway. **(C)** Western blot analysis was performed to assess the expression levels of METTL1 in four glioma cell lines. **(D, E)** Representative EPZ5676 and Ulixertinib dose response curves of U118MG and A172 assessed by CCK-8 assay at 24 h post-treatment.

We appraised the correlation between METTL1 expression and the response to drugs in multiple cancer cell lines. We identified 31 drugs for which METTL1 expression and drug sensitivity were significantly correlated in the GDSC database (**Figure 8A**) **(** 32). The METTL1 expression was positively correlated with the sensitivity to 17 drugs including chromatin histone methylation inhibitors EPZ5676 and GSK343, IGF1R signaling inhibitor Linsitinib, genome integrity inhibitor BIBR-1532, and PI3K/MTOR signaling inhibitor LJI308, etc.; the METTL1 expression was negatively related to drug sensitivity (and this positively correlated with drug resistance) for 14 drugs (**Figure 8A**). Furthermore, the signaling pathways targeted by above drugs were analyzed. Drugs whose sensitivity was related to a high METTL1 expression chiefly targeted the chromatin histone methylation, ERK-MAPK and WNT signaling pathways. Instead, drugs whose sensitivity was bound up with a low METTL1 expression targeted the cell cycle, apoptosis, and protein stability and degradation signaling pathways (**Figure 8B**). Together, these results imply that METTL1 expression is associated with drug sensitivity. In this way, METTL1 is likely to serve as a potential biomarker for designing effective treatment strategies.

We uncovered that A172 had lowest METTL1 expression while U118MG had highest METTL1 expression (**Figure 8C**). CCK-8 assays demonstrated high-METTL1-expression U118MG cells were strikingly more sensitive to chromatin histone methylation inhibitor EPZ5676 and ERK-MAPK inhibitor Ulixertinib (**Figures 8D, E**).

## Discussion

METTL1 was an infrequent methyltransferase to be proved to drive oncogenic transformation by forming m^7^G on tRNA (14). Although studies of METTL1 have been limited, overexpression of METTL1 could drive tumor progression which was reported in hepatocellular carcinoma, colon cancer, intrahepatic cholangiocarcinoma, and lung cancer (9–13). Immunotherapy generally shows a low response rate because of the tumor immune microenvironment (15, 16). Therefore, more research on METTL1 in the fields of TME, immune cells, immunomodulators, immunomodulators and immunotherapeutic response is imminent. In this study, we aim to gain more insights into the underlying mechanism of METTL1 and immune-related factors in pan-cancer research. In the beginning, we investigated the relationship between METTL1 and clinical information and found there were no obvious differences of METTL1 expression among gender, age, tumor stage, status as well as treatment outcome in most cancer types. However, there were significant correlations with METTL1 expression in KIRC. Based on the survival information from the TCGA database, we found that two (LGG and LIHC) of pan-cancer showed a highly consistent relationship between poor prognosis and high METTL1 expression; METTL1 expression in ACC, KIRC, and MESO is significantly related to OS, PFS, and DSS, instead of DFS. In terms of the results that the high expression of METTL1 has various prognostic value in different cancers, we hypothesized that it could be an efficient strategy with clinical benefits to regulate METTL1 therapeutic activity according to different tumor types.

To assess the expression of METTL1 more comprehensively, we conduct METTL1 gene activity based on a gene set containing on hundred genes which were most related to METTL1 expression in pan-cancer. The gene activity was considered to indirectly reflect the difference of METTL1 protein level. Comparing the mRNA expression of METTL1 with METTL1 activity score, we found that the transcription value partially matched the METTL1 activation in some cancers (TGCT, DLBC, UVM, ACC, UCS, SKCM, STAD, and KIRC), indicating that mRNA expression of METTL1 could reveal METTL1 activation in these cancers. However, there was an inconsistency between the activity and expression of METTL1 in other cancers (LUSC, COAD, LUAD as well as KICH). This could be attributed to the protein metabolism and post transcriptional protein level modification.

In addition, we also analyzed the significance of METTL1 on prognosis, and explored its mechanism through gene function enrichment analysis among pan-cancer. GSEA demonstrated that genes from proliferation and metastasis-relevant pathways were more likely to be enriched in the high METTL1 expression cohorts of KIRC, LGG, as well as LIHC.

The TME contains various cells including abundant infiltrating immune cells (33).To explore the probable value of METTL1 moreover, the association between immune cells infiltration and METTL1 was investigated. We observed a marked correlativity between METTL1 and regulatory T cell content in BRCA, KIRP, KIRC, LUAD, LIHC, PRAD, and SARC. In TGCT, a negative association was exhibited in resting mast cells, M2 macrophages, and M0 macrophages and a positive relevancy was evident in activated memory CD4+ T cell, CD8 T cell, follicular helper T cell, and naive B cell. Nevertheless, no definite correlation was discovered between METTL1 and immune cells in DLBC, GBM, and UVM. For diverse immune inhibitors, KDR and CD274 demonstrated the most significant negative relationship with METTL1 among nearly all cancers. Among MHC molecules and immune stimulators, most of the biomarkers exhibited a negative association with METTL1, excluding KIRP, LGG, and TGCT. The findings contribute to the discovery of innovative mechanisms of immunotherapy.

By way of addition, in this study, three ICB therapeutic biomarkers (MSI, TMB, as well as CD274) showed a significant correlation with METTL1 among a few cancers. Patients with high TMB status present acceptable clinical responses to ICB therapy because of the more neoantigens to form (34). Given by inadequate DNA mismatch repair, MSI is defined as a potential prognosticative biomarker for ICB therapy (35). METTL1 was negatively related to MSI and TMB in COAD, nevertheless it was positively correlated with the markers in HNSC, KICH, PRAD, and STAD, which manifested that METTL1 might possess an effect on the ICB therapeutic response in HNSC, KICH, PRAD, as well as STAD. Besides, the correlation between the ICB therapeutic response and METTL1 was explored. The METTL1 expression in the no/limited response group were importantly lower than those in the response group of anti-PD-L1 cohort, insinuating that METTL1 expression can reflect the sensitivity to ICB therapy. However, no significant differences were found in two anti-PD-1 cohorts. To be honest, we only analyzed three relevant cohorts in current study, which was hard to estimate the value of METTL1 to predict the immunotherapeutic response comprehensively. We hypothesize that METTL1 may influence the ICB therapeutic response *via* targeting other immune checkpoints including T cells Ig and ITIM domain (TIGIT) or cytotoxic T lymphocytes associated protein 4 (CTLA-4).

Furthermore, we concluded that drugs whose sensitivity was correlative with a high METTL1 expression mainly targeted the chromatin histone methylation, ERK-MAPK and WNT pathways. Conversely, the drug whose sensitivity was related to a low METTL1 expression targeted the cell cycle, apoptosis, and protein stability and degradation signaling pathways. Together, these results indicated that METTL1 expression is correlated with drug sensitivity. METTL1 expression may be a significant biomarker for projecting rational therapy strategies.

In addition, we evaluated the potential therapeutic effects of METTL1. METTL1 expression was related with resistance to drugs targeting cell cycle, apoptosis, protein stability as well as degradation signaling pathways, and with sensitivity to drugs targeting the chromatin histone methylation, ERKMAPK and WNT pathways. These results demonstrated that patients with higher METTL1 benefit from drugs targeting the chromatin histone methylation, ERK-MAPK and WNT pathways, instead of cell cycle, apoptosis, and protein stability and degradation signaling pathways. Thus, METTL1 expression might be regarded as a qualified predictor that can be used to predict the clinical outcome of targeted or chemotherapy therapies.

As far as we know, this is original research that concentrates on the value of METTL1 in multiple cancers (thirty-three types). This study supplies forward-looking view on the significance of METTL1 in cancer immunotherapy and reveals the correlation between essential immunological indicators and METTL1, which might be advantageous to comprehend the potential mechanisms according to immune system and METTL1.

## Conclusion

This study is instrumental in appraising the ICB therapeutic value of METTL1 among pan-cancer. Increased METTL1 expression was related to adverse prognosis in 14 cancers, especially LGG and LIHC, as well as with high immune infiltration of regulatory T cell, resting mast cell, and M2 macrophage. Therefore, METTL1 is important for immune cell infiltration and may represent a distinct biomarker. We are confident that these findings may provide suggestions for experimentation and have implications for clinical treatment.

## Data Availability Statement

The datasets presented in this study can be found in online repositories. The names of the repository/repositories and accession number(s) can be found below: TCGA: https://xena.ucsc.edu/; GEO: https://www.ncbi.nlm.nih.gov/geo/ under the accession numbers GSE78220 and GSE67501; GDSC: https://www.cancerrxgene.org/; CCLE: https://portals.broadinstitute.org/ccle/.

## Ethics Statement

The studies involving experiments were reviewed and approved by the Ethics Committee of the Qilu Hospital (Jinan, China) and performed in accordance with the relevant guidelines and regulations; the patient data were acquired from the publicly available datasets, and written informed consent was given.

## Author Contributions

ZG, JX, PZ, and GL designed this work. ZG, JX, ZZ, and YF integrated and analyzed the data. ZG, JX, ZZ, HX, and XG wrote this manuscript. ZG, JX, YF, SW, LD, and RZ edited and revised the manuscript. All authors read and approved this manuscript.

## Funding

This work was supported by grants from the National Natural Science Foundation of China (Nos. 81874083; 82072776; 82072775; 81702468; 81802966; 81902540; 81874082; 81472353), Natural Science Foundation of Shandong Province of China (Nos. ZR2019BH057; ZR2020QH174; ZR2021LSW025), the Jinan Science and Technology Bureau of Shandong Province (2021GXRC029), Key clinical Research project of Clinical Research Center of Shandong University (2020SDUCRCA011) and Taishan Pandeng Scholar Program of Shandong Province (No. tspd20210322).

## Conflict of Interest

The authors declare that the research was conducted in the absence of any commercial or financial relationships that could be construed as a potential conflict of interest

## Publisher’s Note

All claims expressed in this article are solely those of the authors and do not necessarily represent those of their affiliated organizations, or those of the publisher, the editors and the reviewers. Any product that may be evaluated in this article, or claim that may be made by its manufacturer, is not guaranteed or endorsed by the publisher.

## Supplementary Material

Supplementary Figure S1 Correlation between METTL1 and clinical traits. **(A)** The relationship between METTL1 expression and gender. **(B)** The relationship between METTL1 expression and treatment response. **(C)** The relationship between METTL1 activity and gender. **(D)** The relationship between METTL1 activity and treatment response. **(E)** The mean expression as well as activity value of METTL1 in pan-cancer.

Supplementary Figure S2 Kaplan-Meier curves showing the survival information of different METTL1 expression level in pan-cancer.

Supplementary Figure S3 ROC curve showing sensitivity of METTL1 as a marker to predict prognosis in pan-cancer.

Supplementary Figure S4 Univariate Cox regression analyses for METTL1 activity in pan-cancer. Forest plot visualizing the association of METTL1 activity and OS, DFS, DSS as well as PFS respectively among pan-cancer. HR value > 1 represents risk factor, whereas HR value < 1 represents favorable factor.

Supplementary Figure S5
**(A)** Transwell assay performed in U251 transfected si-METTL1#1, si-METTL1#2, vector and ov- METTL1 cells (scale bar=100 µm). **(B)** Transwell assays showed the ability of growth media of different CNE2 cells to recruit macrophages (scale bar=100 µm). **(C)** Multivariate Cox regression analysis demonstrated that the expression of METTL1 was independent risk factor in LGG. **(D)** Multivariate Cox regression analysis in LIHC.

Supplementary Figure S6 Analysis of METTL1 activity related immune characteristics. **(A)** Heatmap visualized the relationship between METTL1 activity and immune score, stromal score, tumor purity as well as ESTIMATE score in pan-cancer. **(B)** Heatmap showed correlation between METTL1 activity and 22 immune cell infiltration value obtained by CIBERSORT algorithm among pan-cancer. **(C)** Heatmap exhibited the relationship between METTL1 activity and stemness indices among pan-cancer. The upper half of each grid exhibited the p value and the lower half exhibited the correlation coefficient.

Supplementary Figure S7
**(A-C)** The relationship between the METTL1 expression and immune inhibitors **(A)**, immune stimulators **(B)** as well as MHC molecules **(C)** respectively.

Supplementary Figure S8 Role of METTL1 expression in response to ICB therapy. **(A)** The proportion of patients with immune cell infiltration phenotype in low or high METTL1 expression groups. **(B)** The column chart showed the proportion of patients without platinum therapy with low or high METTL1 expression who responded to PD-L1 block immunotherapy. **(C)** Kaplan-Meier curve showed the survival of the high and low METTL1 expression patient without platinum therapy groups in anti-PD-L1 immunotherapy cohort. **(D, E)** Differences of METTL1 expression between patients with different immunotherapy responses in the GSE78220 and GSE67501 cohorts respectively. **(F, G)** Differences of METTL1 activity between patients with different immunotherapy responses in the GSE78220 and GSE67501 cohorts respectively.

Supplementary Figure S9 GSEA results based on Hallmark dataset among pan-cancer.
