# Supplementary figures and images for: A Comprehensive Analysis of METTL1 to Immunity and Stemness in Pan-Cancer
Source: Front Immunol. 2022 Mar 31;13:795240. doi: 10.3389/fimmu.2022.795240 (PMC9008260; doi:10.3389/fimmu.2022.795240)

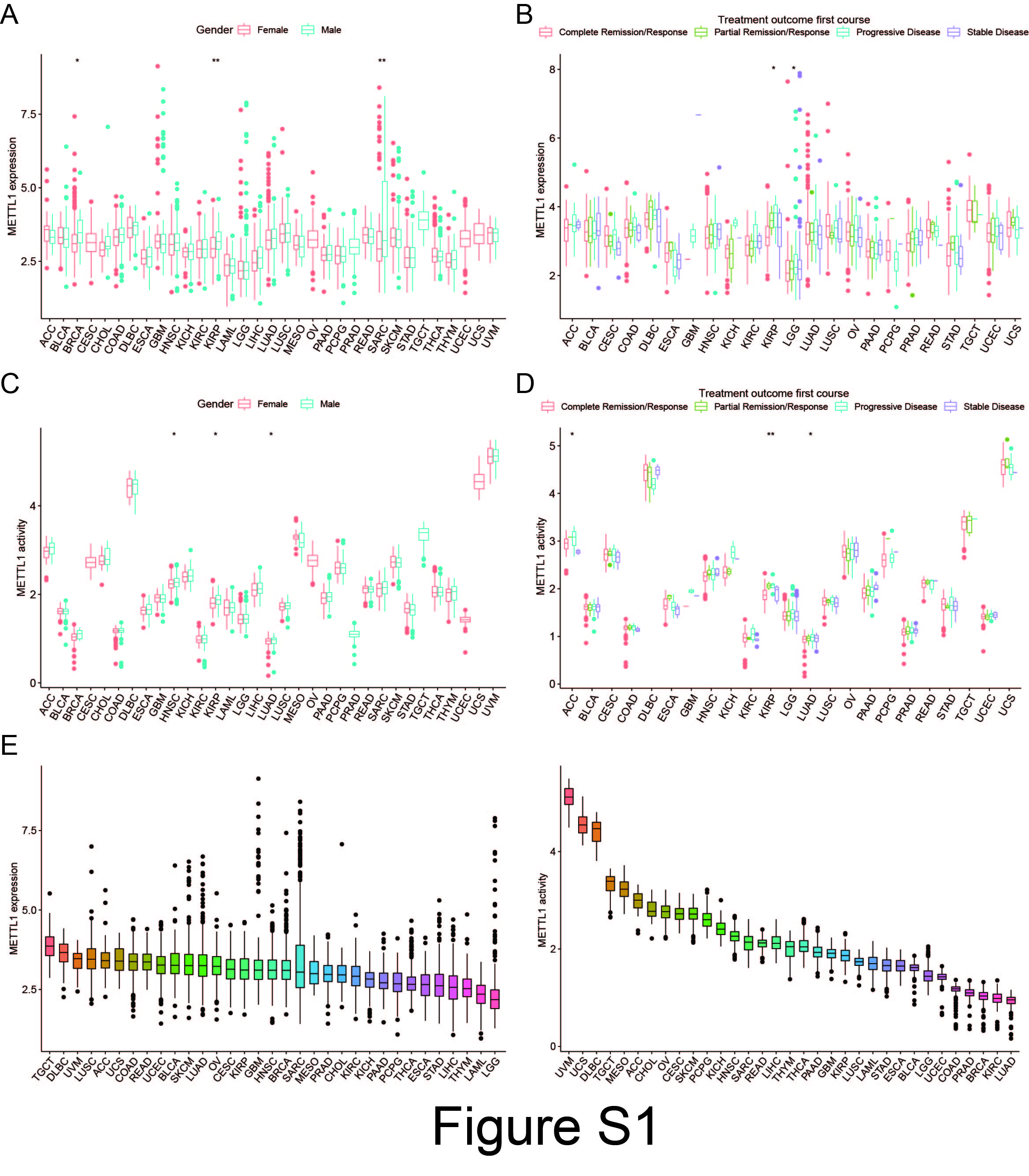

Supplement: Supplementary Figure S1 — Correlation between METTL1 and clinical traits. (A) The relationship between METTL1 expression and gender. (B) The relationship between METTL1 expression and treatment response. (C) The relationship between METTL1 activity and gender. (D) The relationship between METTL1 activity and treatment response. (E) The mean expression as well as activity value of METTL1 in pan-cancer. [file Image_1.jpeg]

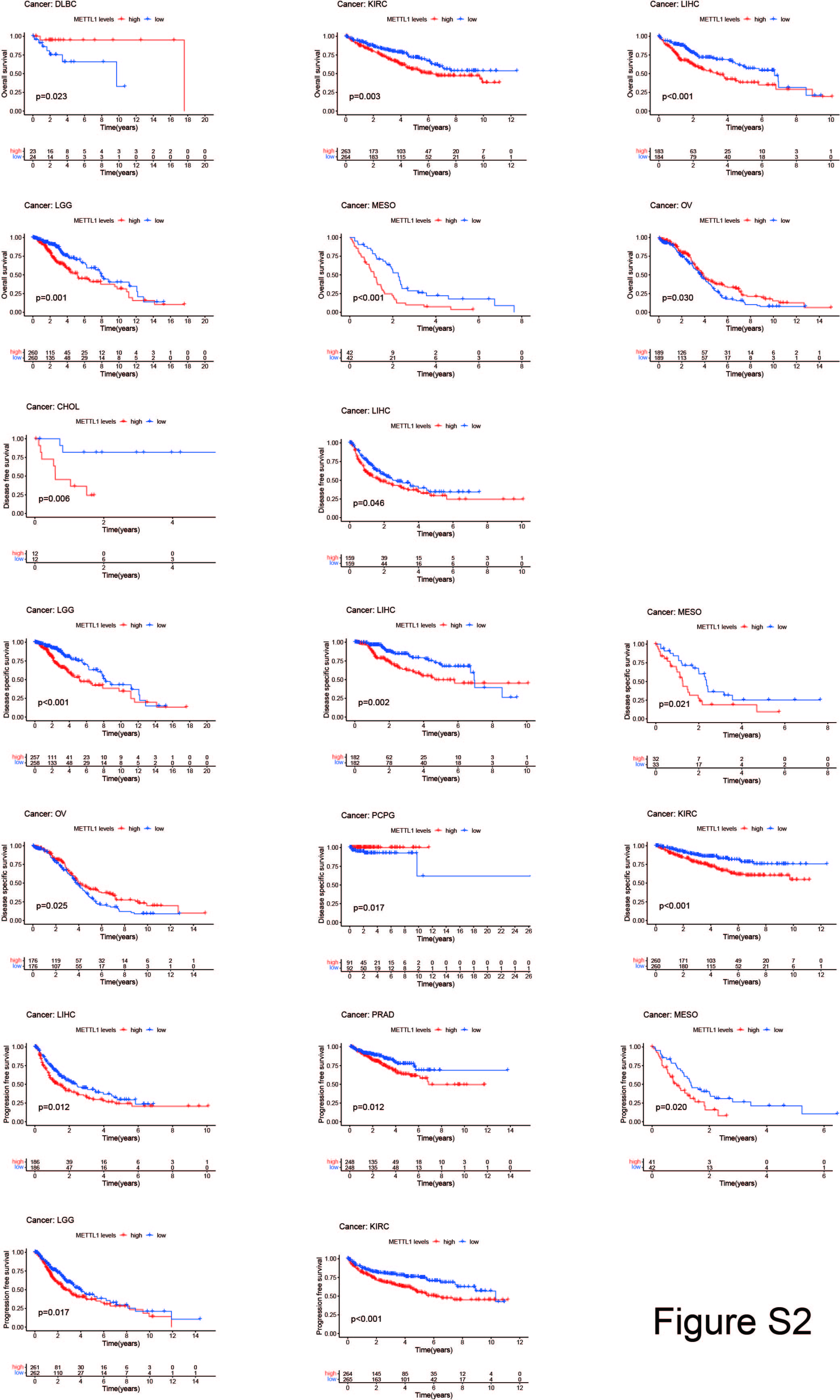

Supplement: Supplementary Figure S2 — Kaplan-Meier curves showing the survival information of different METTL1 expression level in pan-cancer. [file Image_2.jpeg]

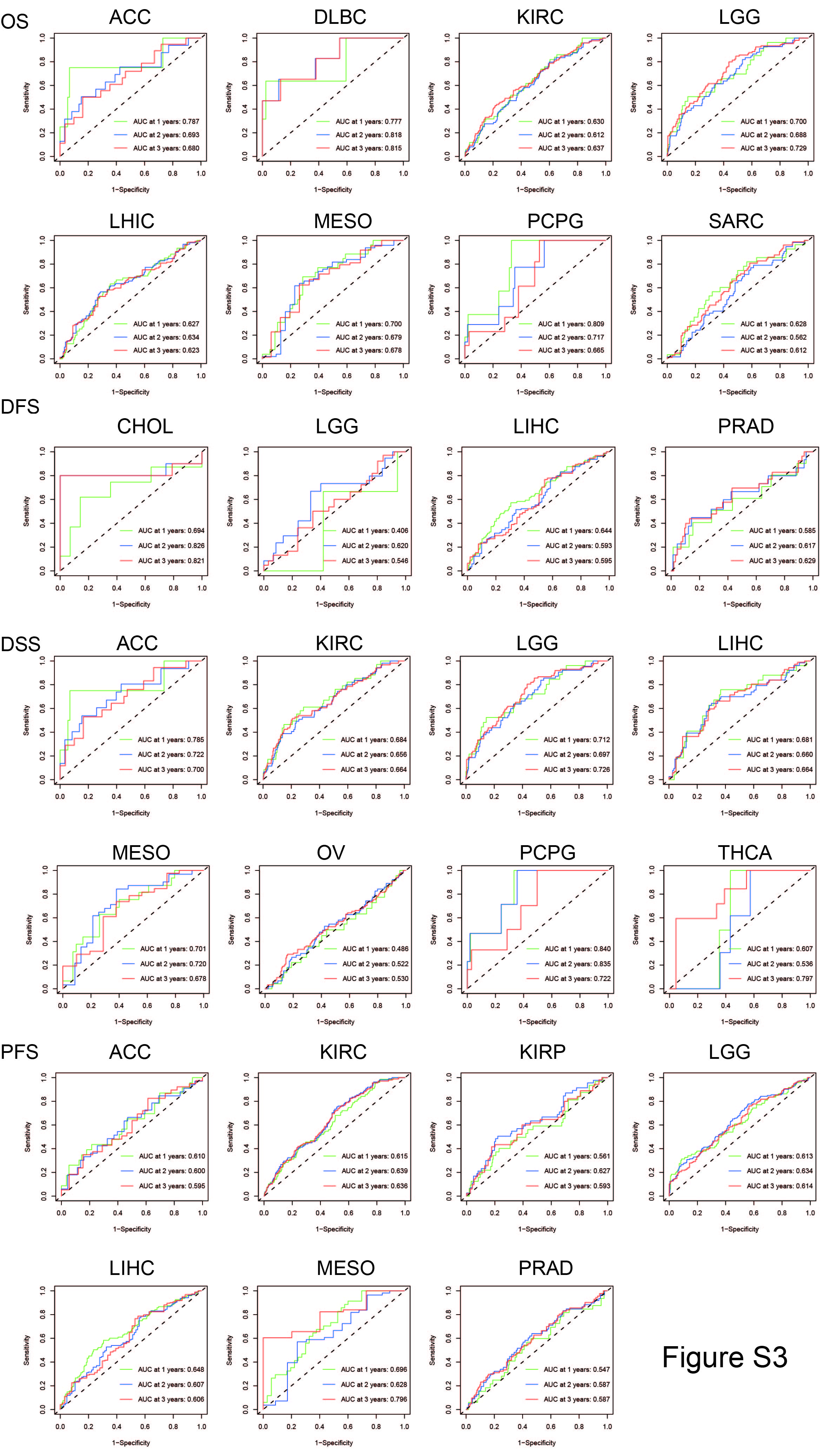

Supplement: Supplementary Figure S3 — ROC curve showing sensitivity of METTL1 as a marker to predict prognosis in pan-cancer. [file Image_3.jpeg]

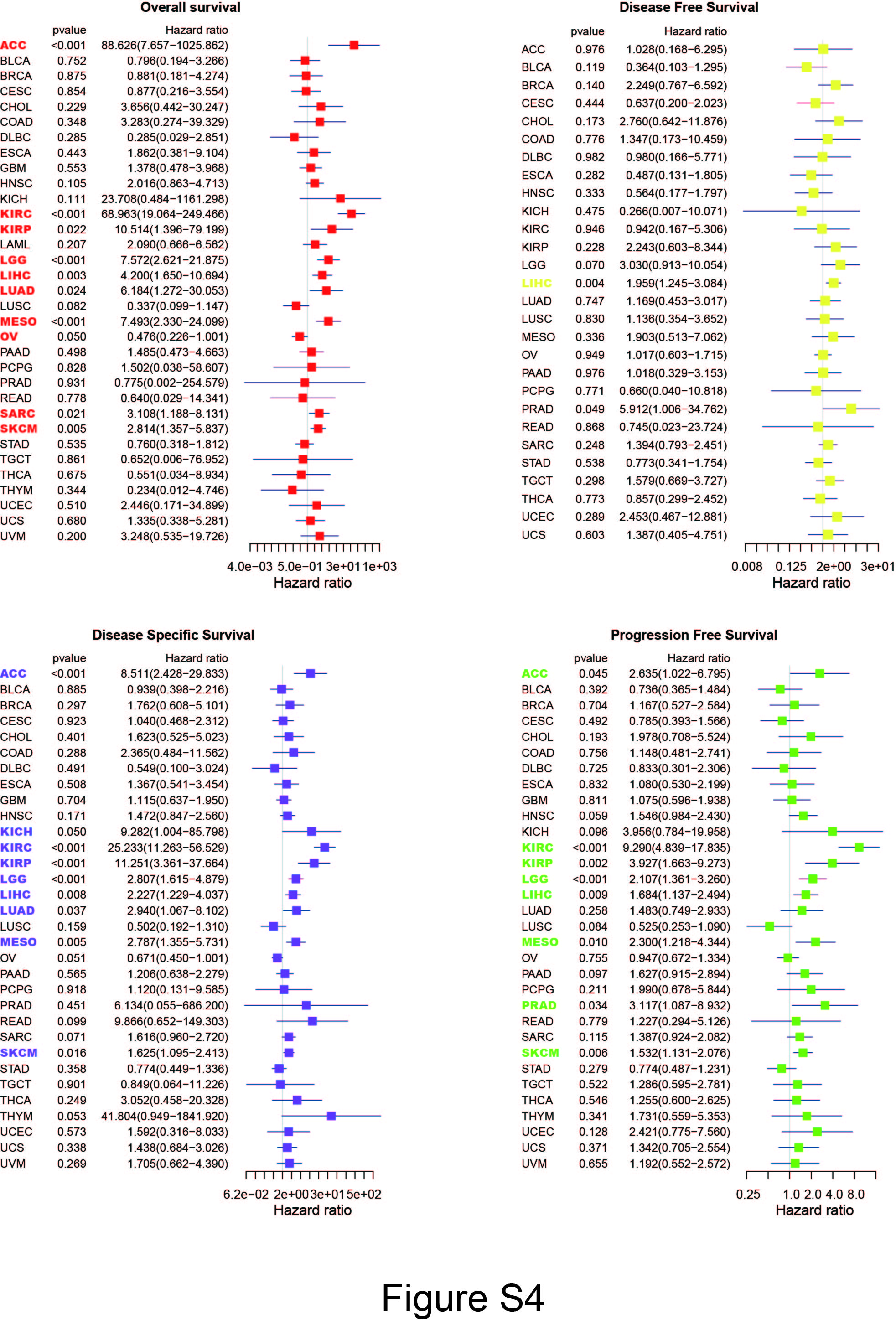

Supplement: Supplementary Figure S4 — Univariate Cox regression analyses for METTL1 activity in pan-cancer. Forest plot visualizing the association of METTL1 activity and OS, DFS, DSS as well as PFS respectively among pan-cancer. HR value > 1 represents risk factor, whereas HR value < 1 represents favorable factor. [file Image_4.jpeg]

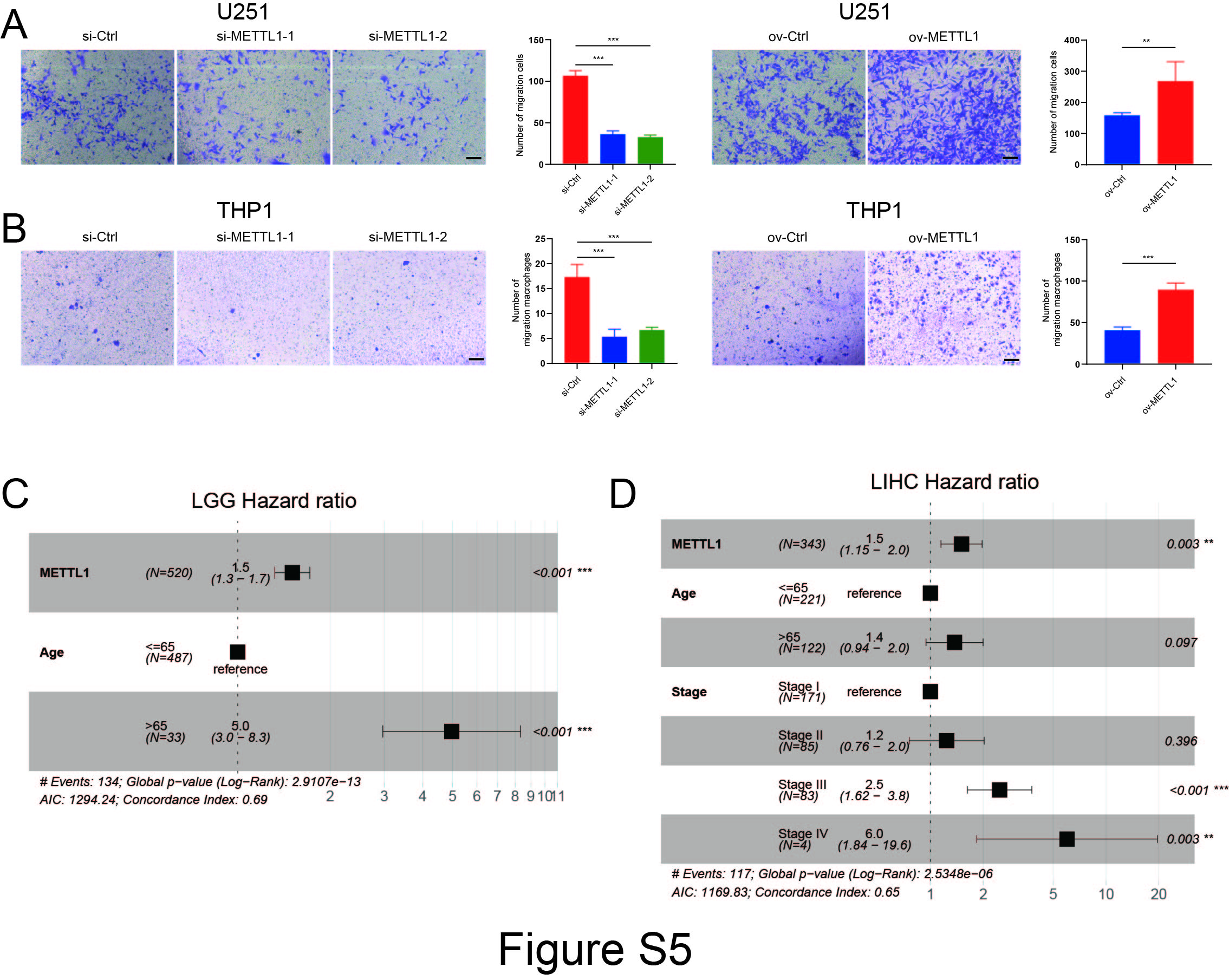

Supplement: Supplementary Figure S5 — (A) Transwell assay performed in U251 transfected si-METTL1#1, si-METTL1#2, vector and ov- METTL1 cells (scale bar=100 µm). (B) Transwell assays showed the ability of growth media of different CNE2 cells to recruit macrophages (scale bar=100 µm). (C) Multivariate Cox regression analysis demonstrated that the expression of METTL1 was independent risk factor in LGG. (D) Multivariate Cox regression analysis in LIHC. [file Image_5.jpeg]

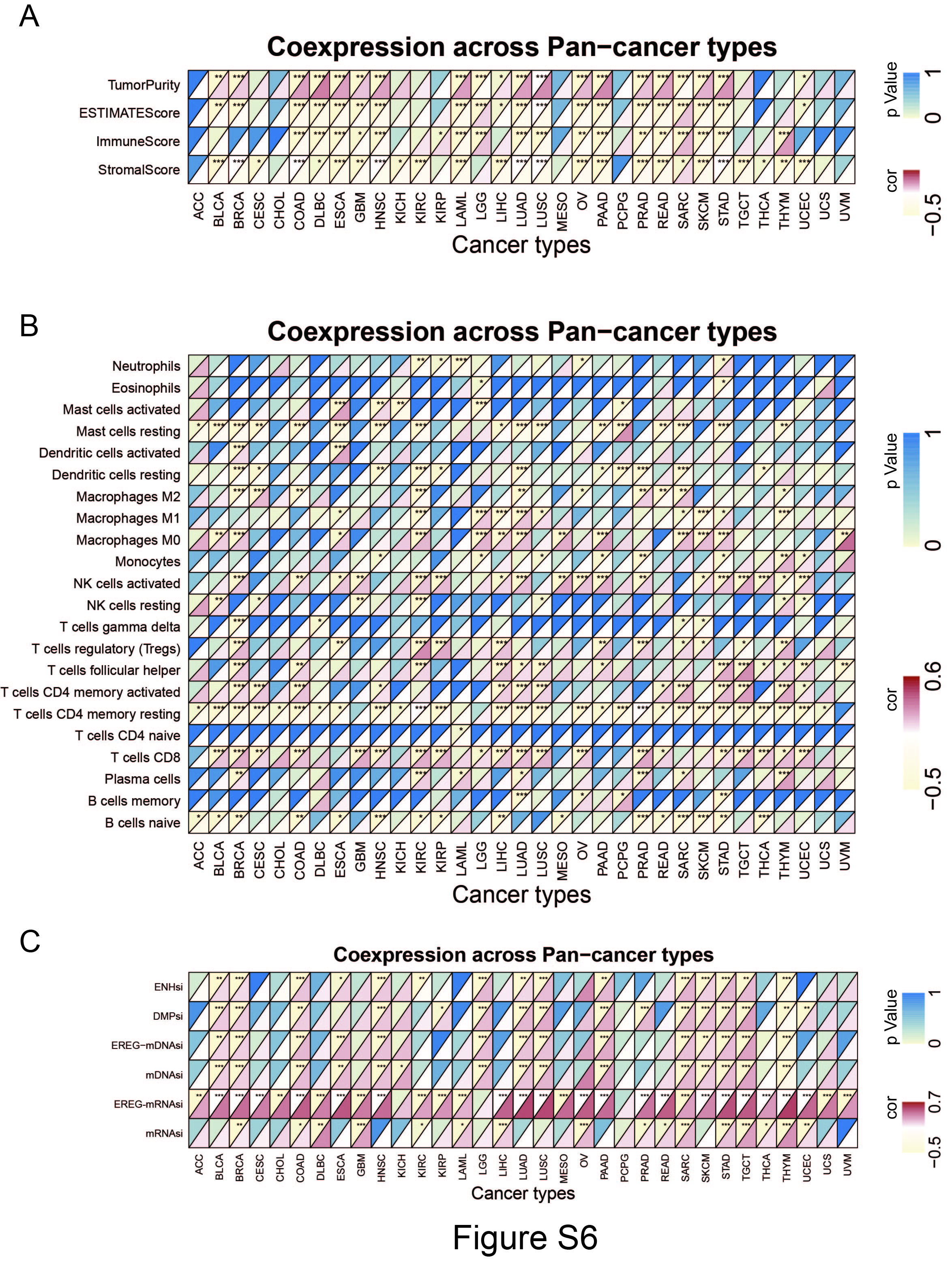

Supplement: Supplementary Figure S6 — Analysis of METTL1 activity related immune characteristics. (A) Heatmap visualized the relationship between METTL1 activity and immune score, stromal score, tumor purity as well as ESTIMATE score in pan-cancer. (B) Heatmap showed correlation between METTL1 activity and 22 immune cell infiltration value obtained by CIBERSORT algorithm among pan-cancer. (C) Heatmap exhibited the relationship between METTL1 activity and stemness indices among pan-cancer. The upper half of each grid exhibited the p value and the lower half exhibited the correlation coefficient. [file Image_6.jpeg]

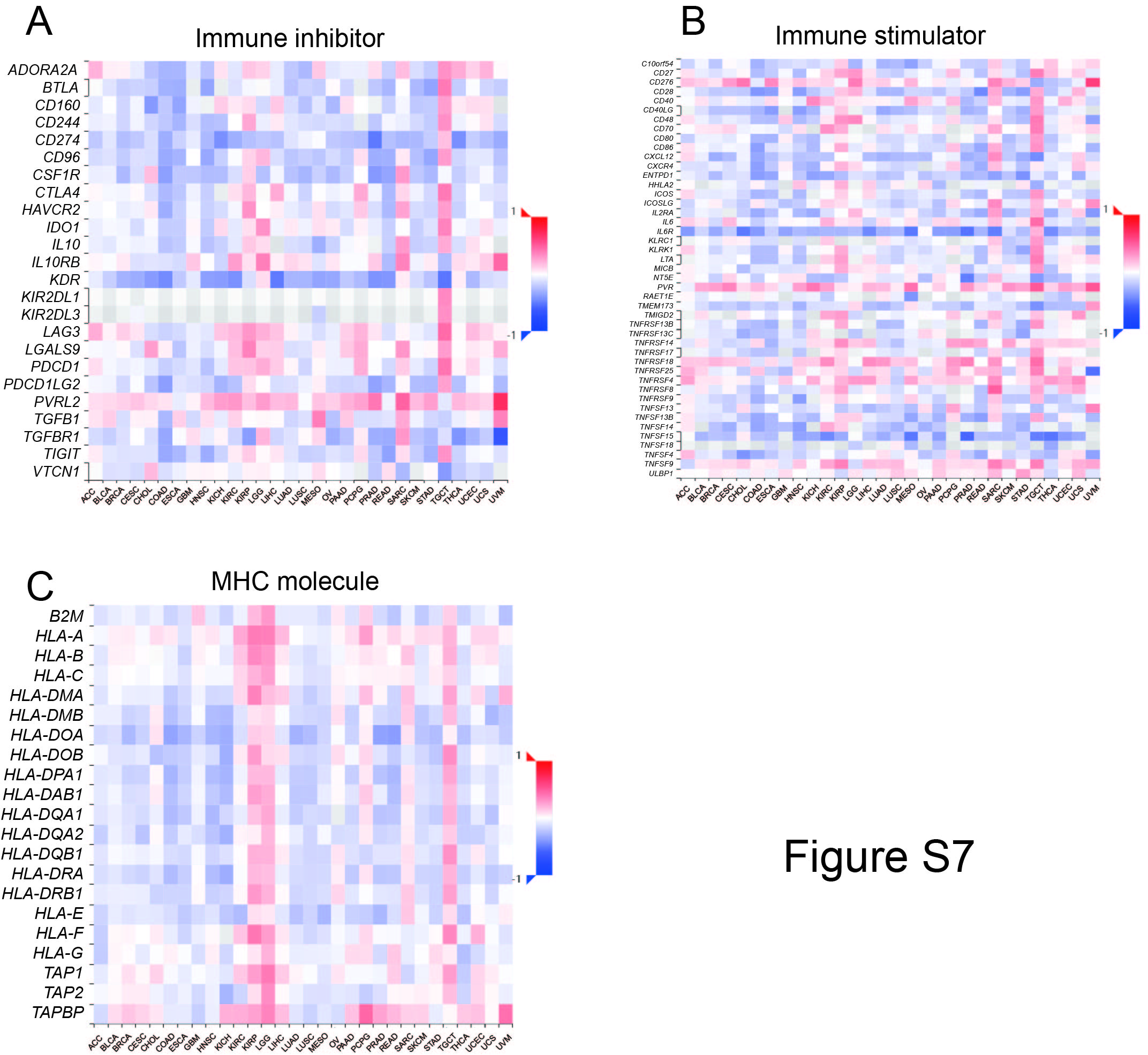

Supplement: Supplementary Figure S7 — (A-C) The relationship between the METTL1 expression and immune inhibitors (A), immune stimulators (B) as well as MHC molecules (C) respectively. [file Image_7.jpeg]

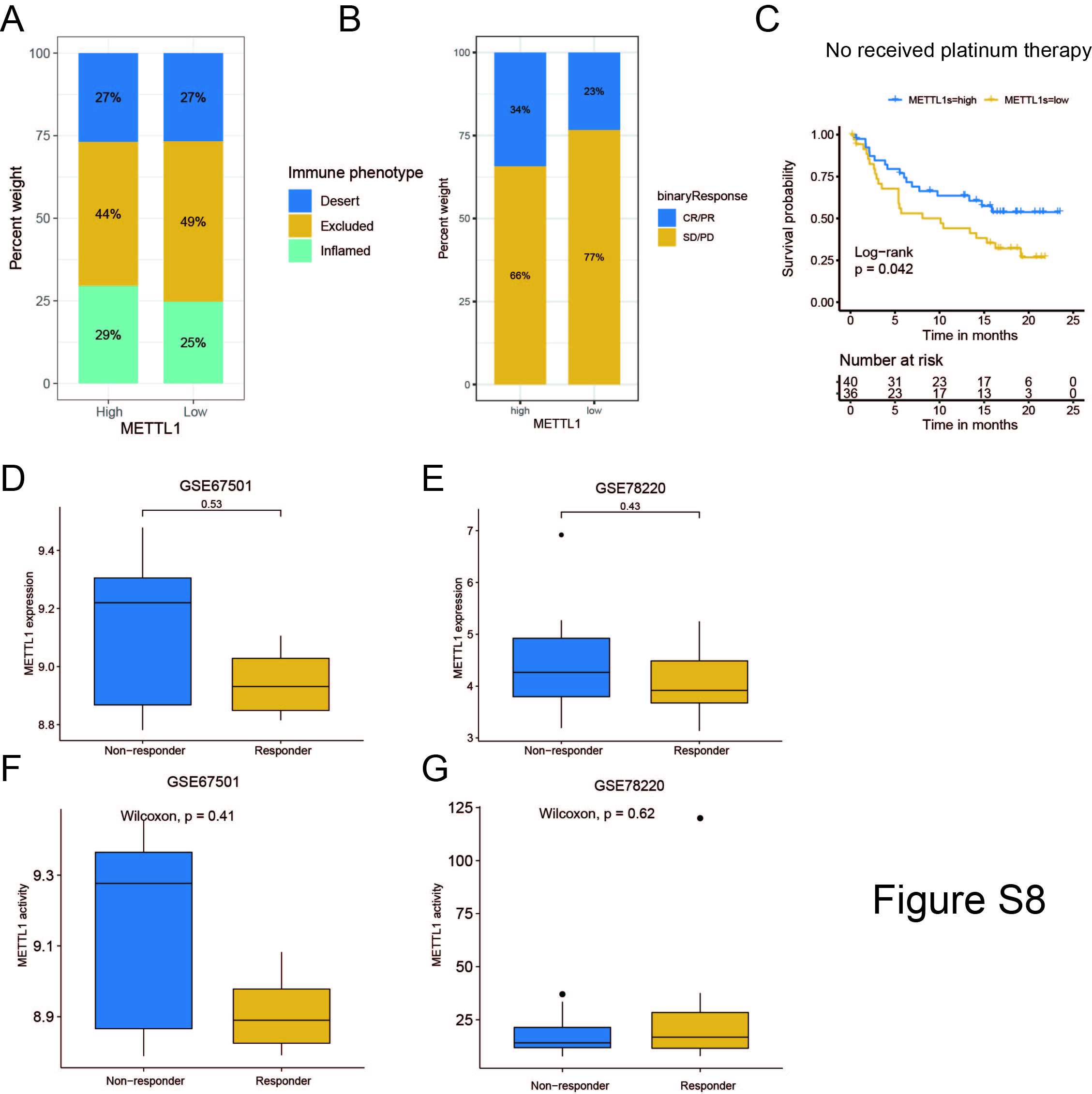

Supplement: Supplementary Figure S8 — Role of METTL1 expression in response to ICB therapy. (A) The proportion of patients with immune cell infiltration phenotype in low or high METTL1 expression groups. (B) The column chart showed the proportion of patients without platinum therapy with low or high METTL1 expression who responded to PD-L1 block immunotherapy. (C) Kaplan-Meier curve showed the survival of the high and low METTL1 expression patient without platinum therapy groups in anti-PD-L1 immunotherapy cohort. (D, E) Differences of METTL1 expression between patients with different immunotherapy responses in the GSE78220 and GSE67501 cohorts respectively. (F, G) Differences of METTL1 activity between patients with different immunotherapy responses in the GSE78220 and GSE67501 cohorts respectively. [file Image_8.jpeg]
