# Supplementary material for: A Comprehensive Analysis of METTL1 to Immunity and Stemness in Pan-Cancer
Source: Front Immunol. 2022 Mar 31;13:795240. doi: 10.3389/fimmu.2022.795240 (PMC9008260; doi:10.3389/fimmu.2022.795240)
Supplement: Supplementary Figure S9 — GSEA results based on Hallmark dataset among pan-cancer. [file Image_9.pdf]

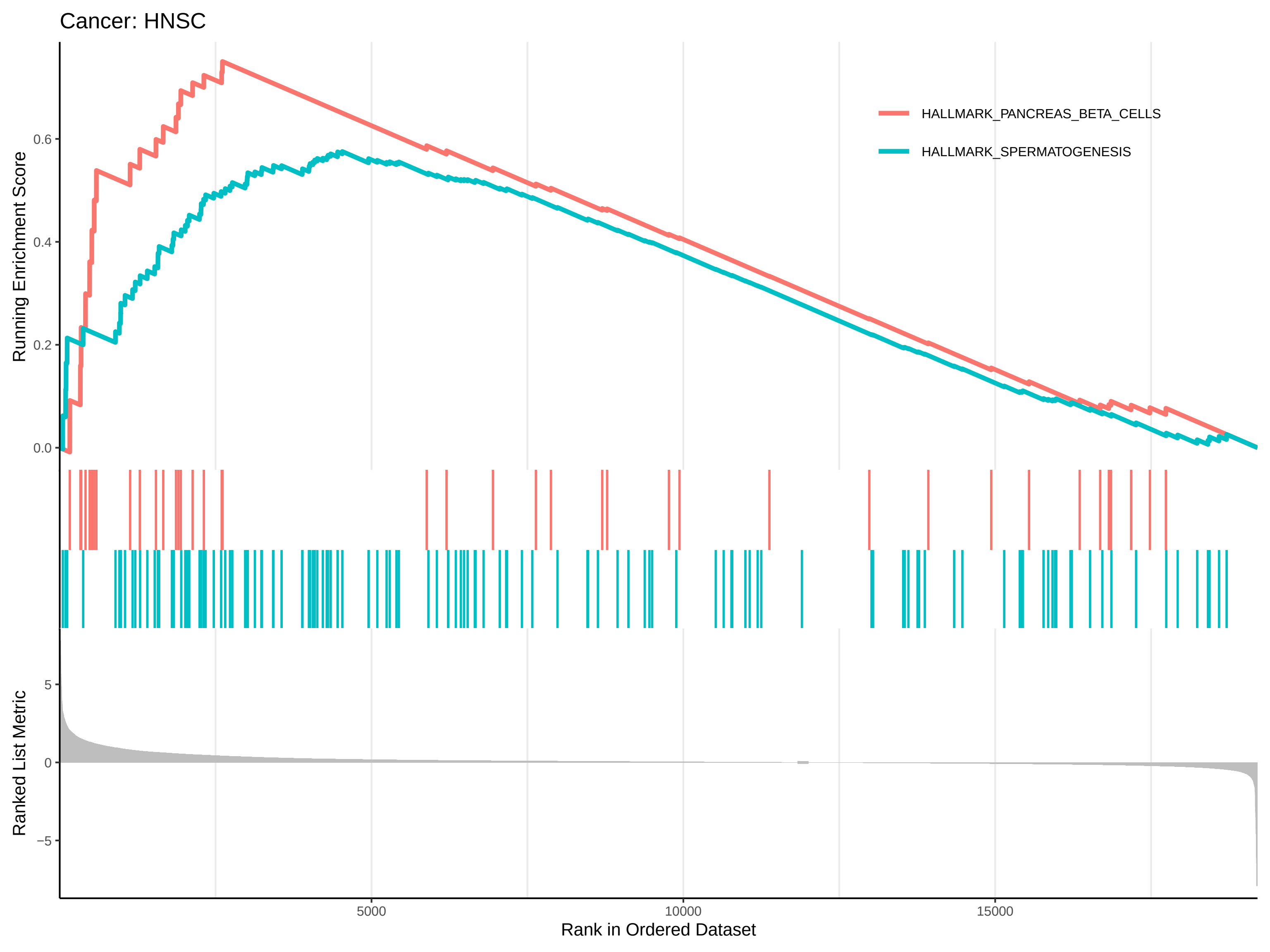

Cancer: HNSC
HALLMARK_PANCREAS_BETA_CELLS
HALLMARK_SPERMATOGENESIS
Running Enrichment Score
0.6
0.4
0.2
0.0
Ranked List Metric
5
0
−5
5000
10000
15000
Rank in Ordered Dataset

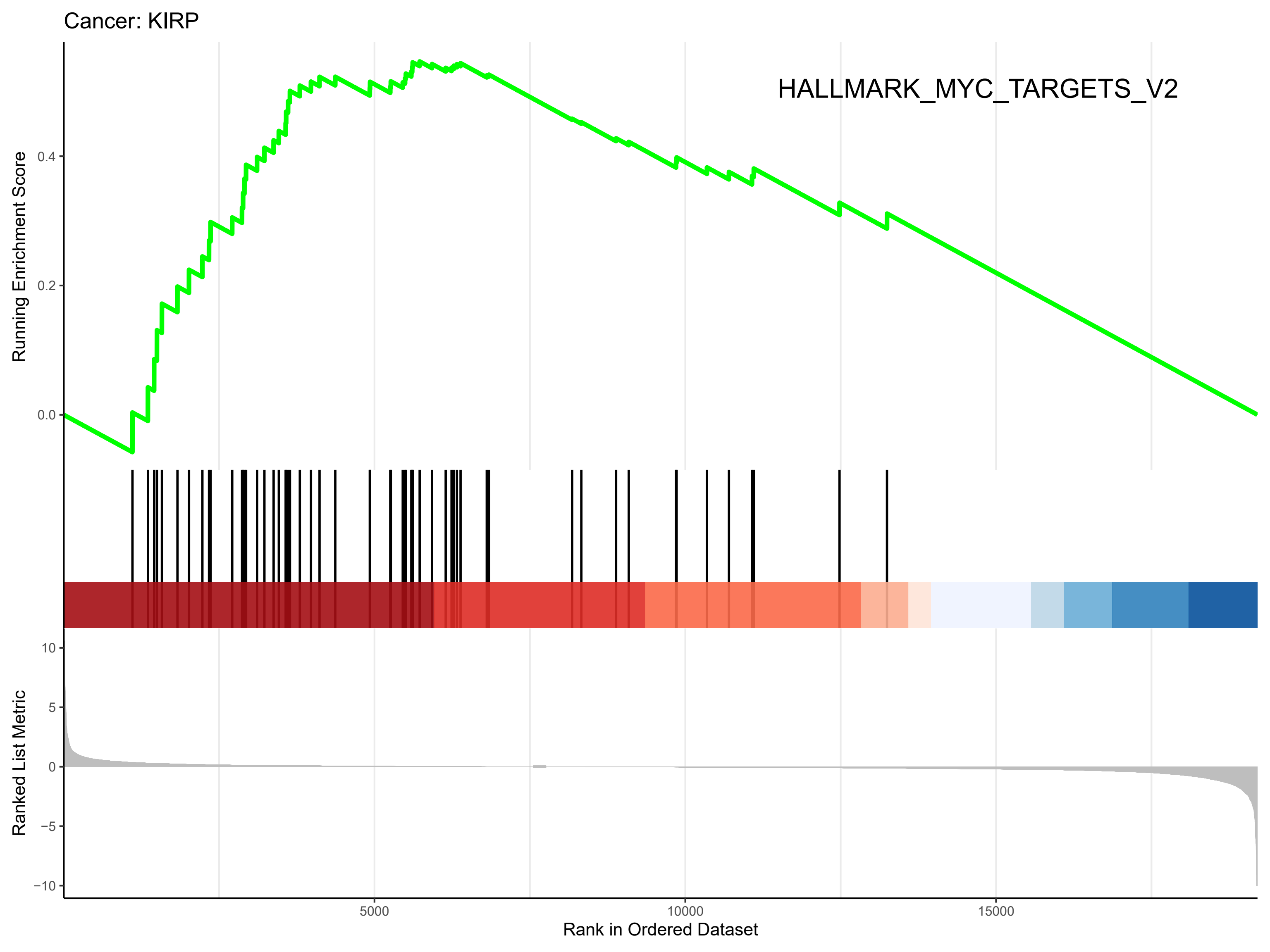
Cancer: KIRP
HALLMARK_MYC_TARGETS_V2
Running Enrichment Score
0.4
0.2
0.0
Ranked List Metric
10
5
0
-5
-10
5000
10000
15000
Rank in Ordered Dataset

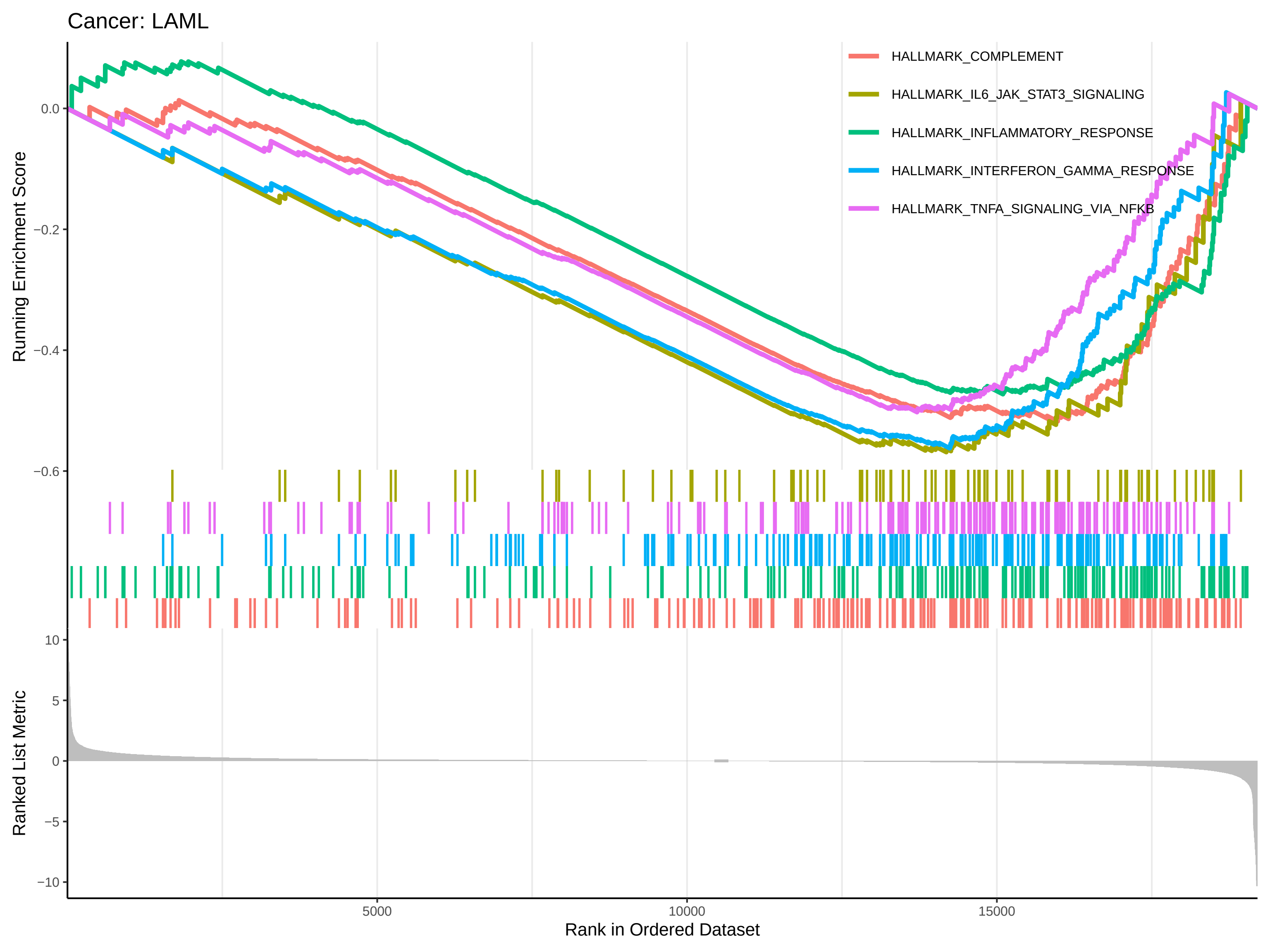

Cancer: LAML
HALLMARK_COMPLEMENT
HALLMARK_IL6_JAK_STAT3_SIGNALING
HALLMARK_INFLAMMATORY_RESPONSE
HALLMARK_INTERFERON_GAMMA_RESPONSE
HALLMARK_TNFA_SIGNALING_VIA_NFKB
Running Enrichment Score
0.0
−0.2
−0.4
−0.6
Ranked List Metric
10
5
0
−5
−10
5000
10000
15000
Rank in Ordered Dataset

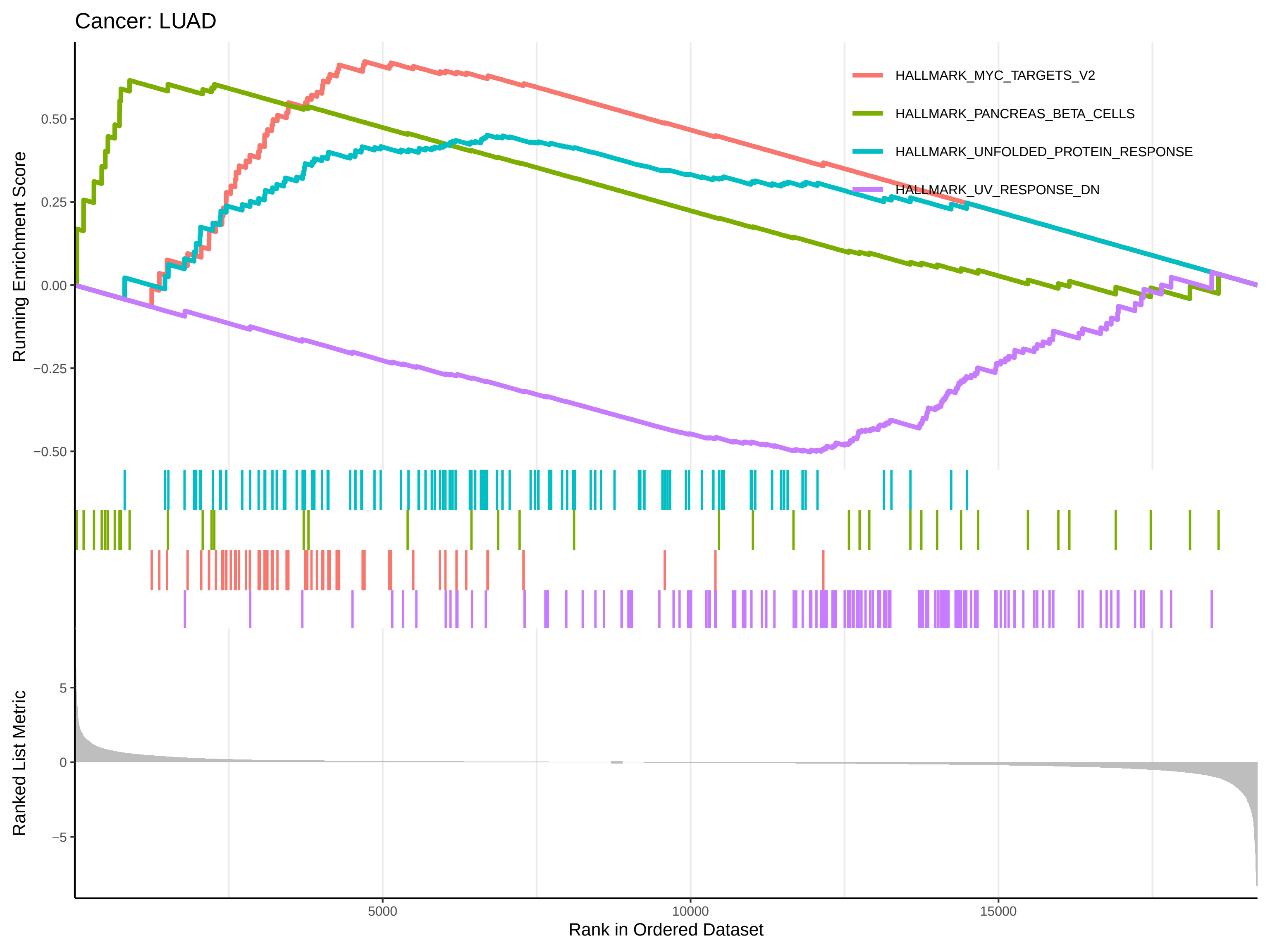

Cancer: LUAD
HALLMARK_MYC_TARGETS_V2
HALLMARK_PANCREAS_BETA_CELLS
HALLMARK_UNFOLDED_PROTEIN_RESPONSE
HALLMARK_UV_RESPONSE_DN
Running Enrichment Score
0.50
0.25
0.00
−0.25
−0.50
Ranked List Metric
5
0
−5
5000
10000
15000
Rank in Ordered Dataset

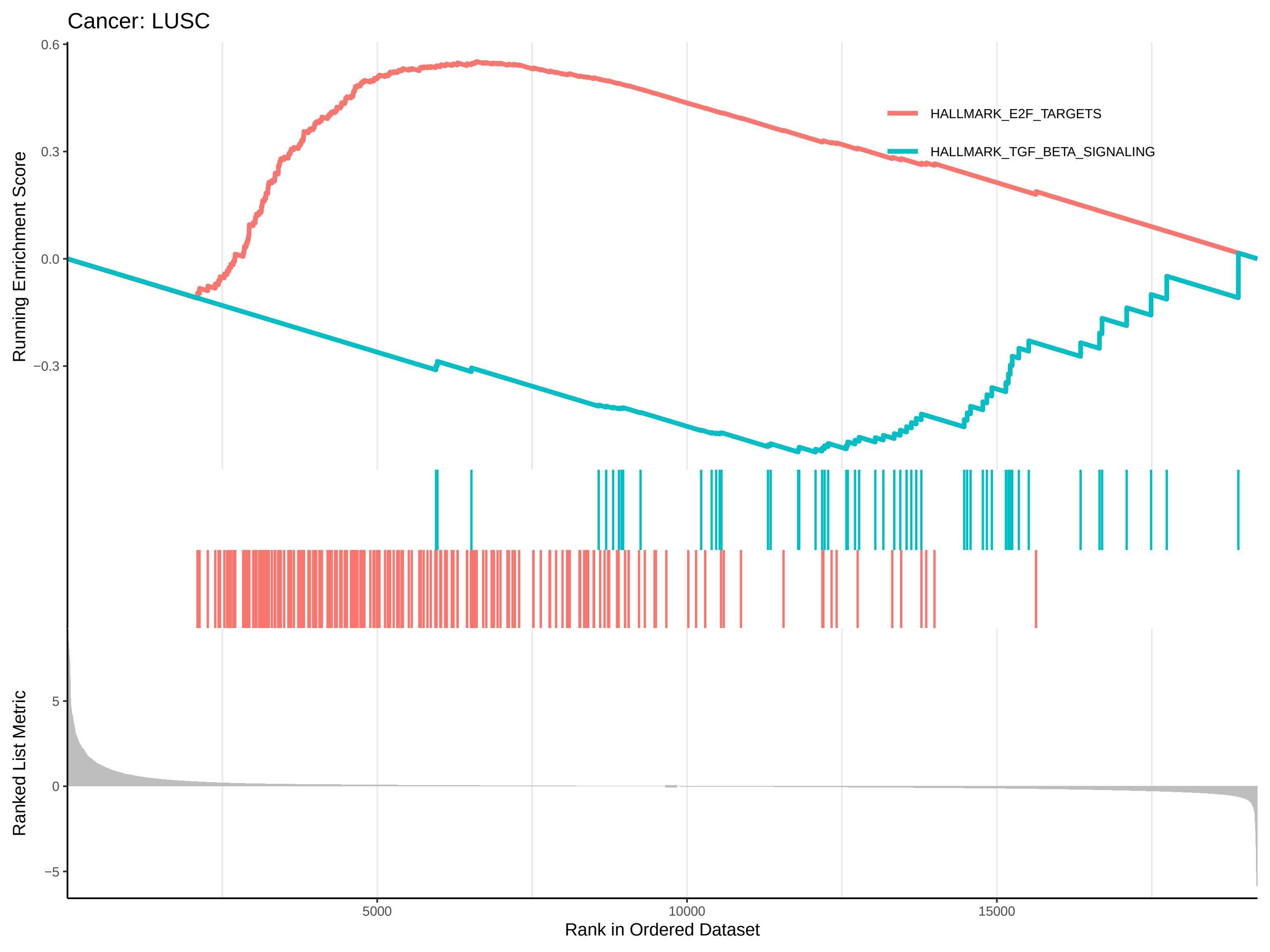
Cancer: LUSC
Running Enrichment Score
0.6
0.3
0.0
−0.3
HALLMARK_E2F_TARGETS
HALLMARK_TGF_BETA_SIGNALING
Ranked List Metric
5
0
−5
5000
10000
15000
Rank in Ordered Dataset

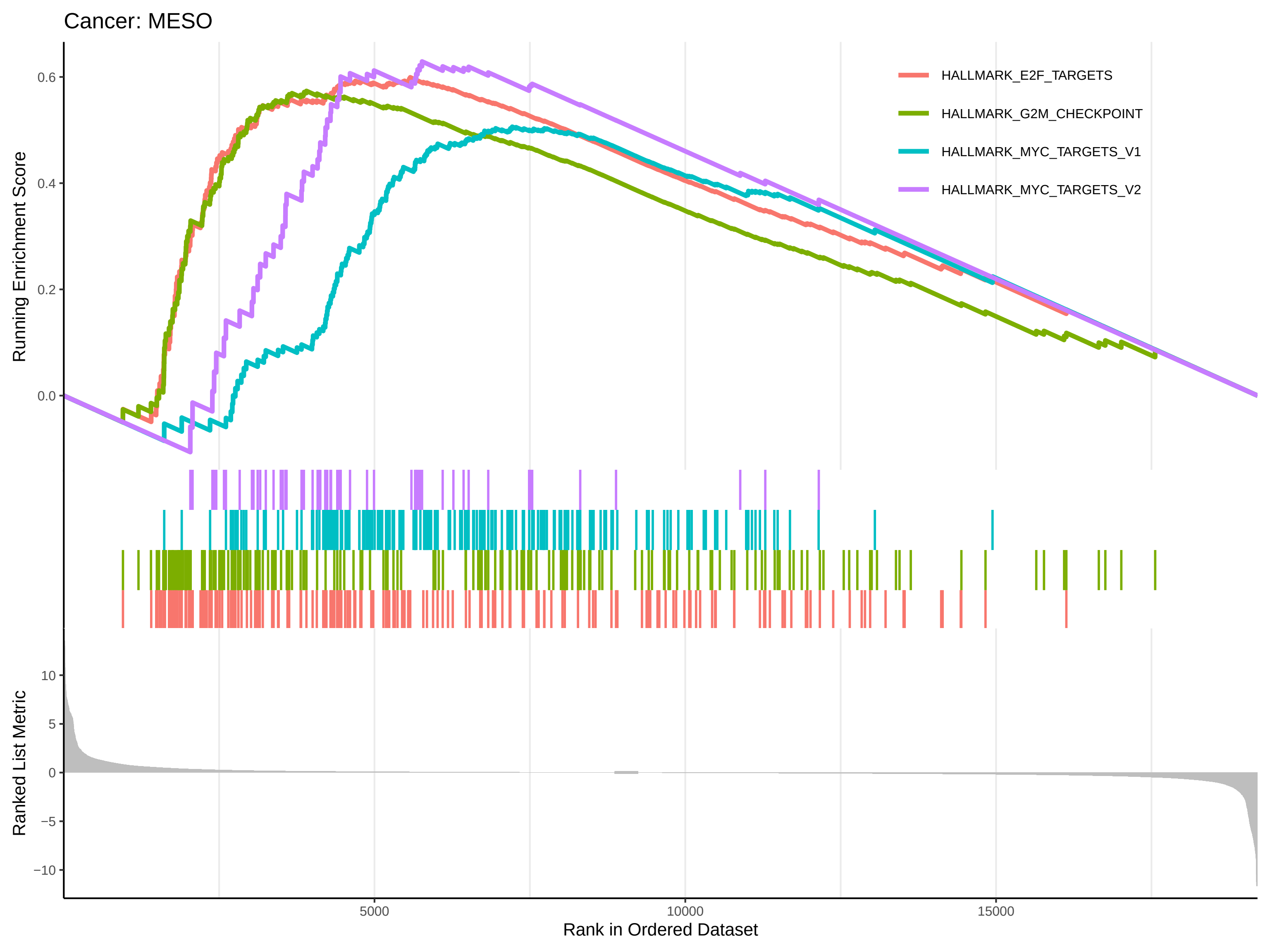

Cancer: MESO
Running Enrichment Score
0.6
0.4
0.2
0.0
HALLMARK_E2F_TARGETS
HALLMARK_G2M_CHECKPOINT
HALLMARK_MYC_TARGETS_V1
HALLMARK_MYC_TARGETS_V2
Ranked List Metric
10
5
0
−5
−10
5000
10000
15000
Rank in Ordered Dataset

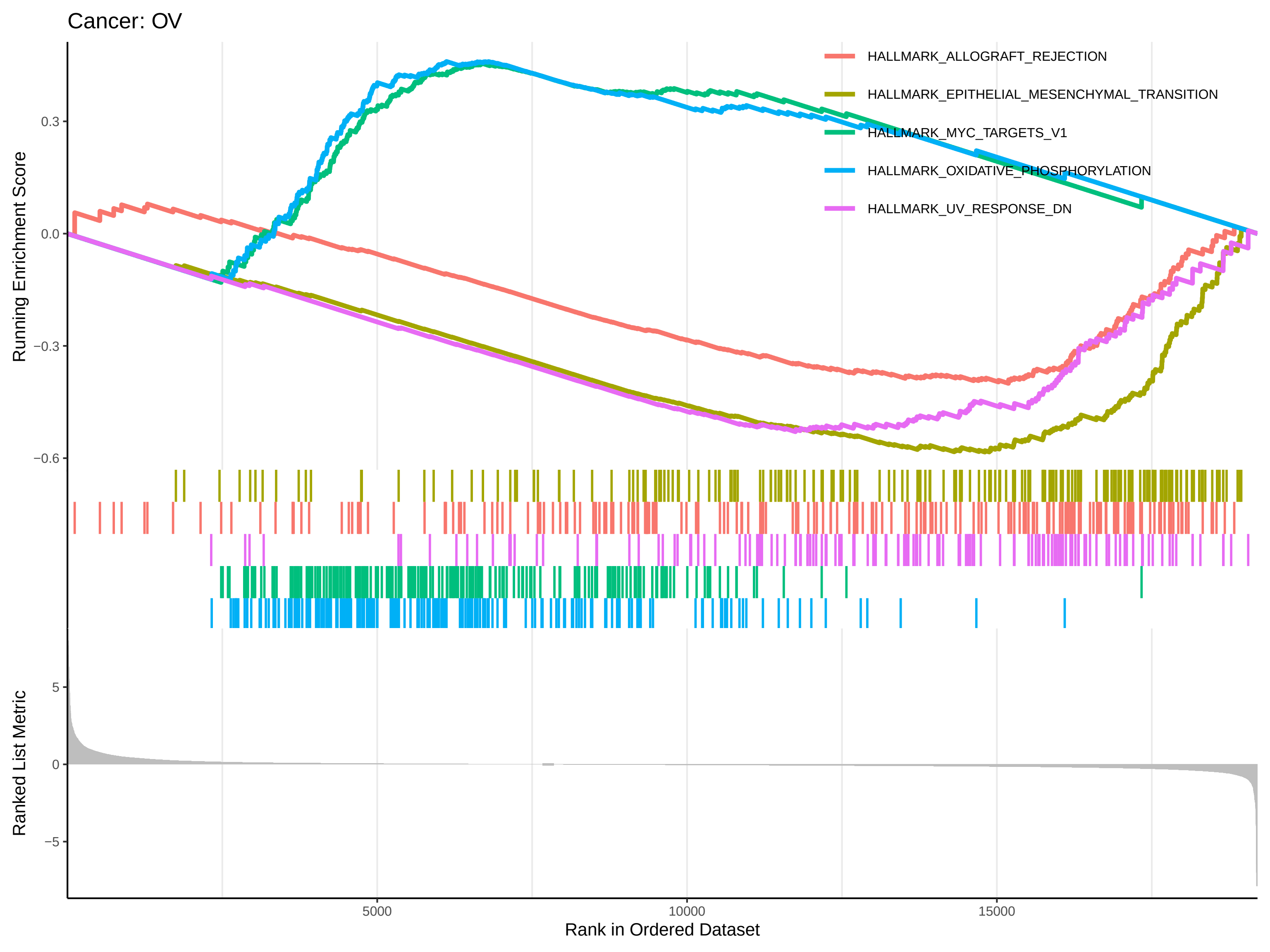
Cancer: OV
Running Enrichment Score
0.3
0.0
−0.3
−0.6
HALLMARK_ALLOGRAFT_REJECTION
HALLMARK_EPITHELIAL_MESENCHYMAL_TRANSITION
HALLMARK_MYC_TARGETS_V1
HALLMARK_OXIDATIVE_PHOSPHORYLATION
HALLMARK_UV_RESPONSE_DN
Ranked List Metric
5
0
−5
5000
10000
15000
Rank in Ordered Dataset

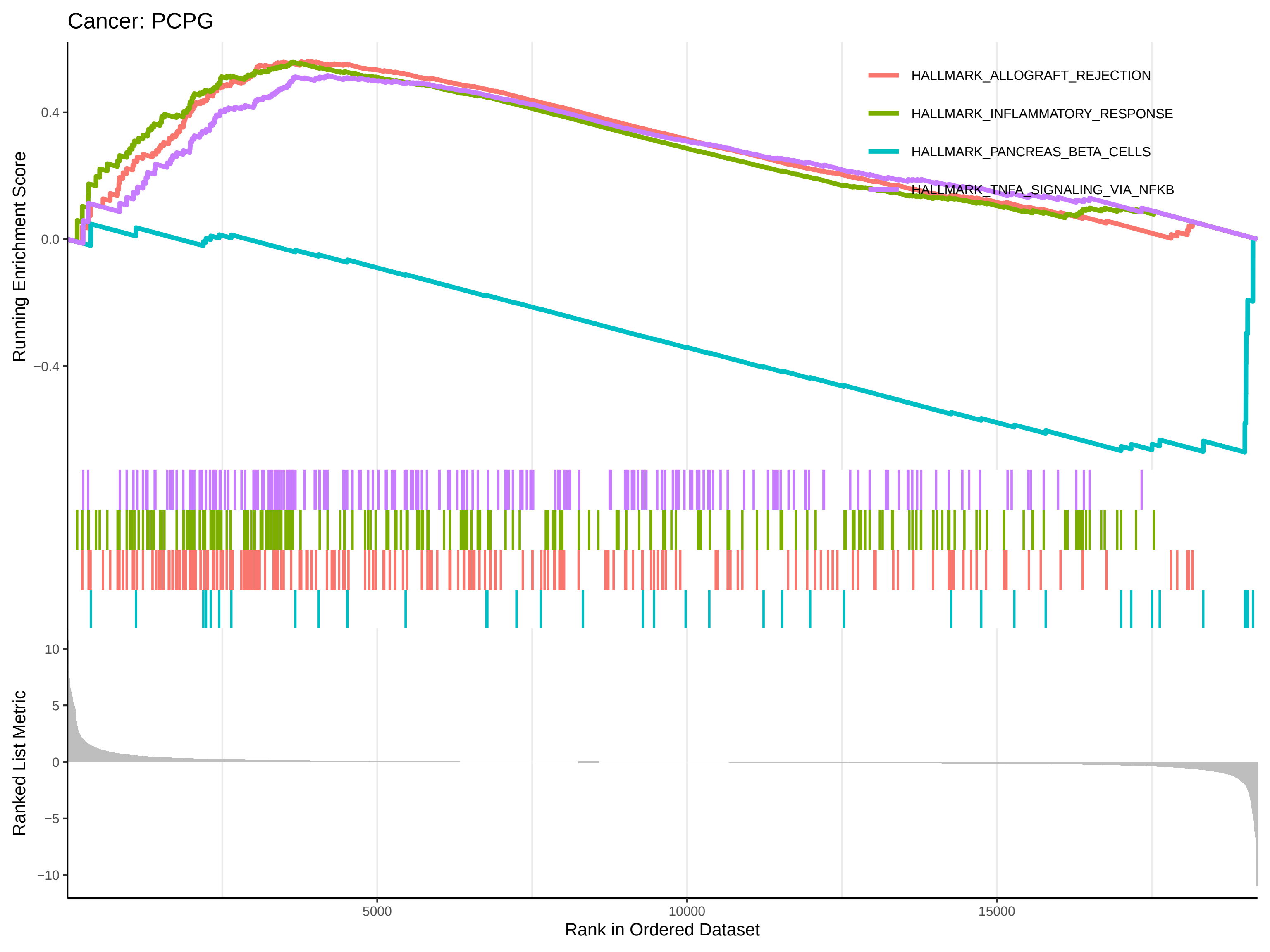

Cancer: PCPG
Running Enrichment Score
0.4
0.0
−0.4
HALLMARK_ALLOGRAFT_REJECTION
HALLMARK_INFLAMMATORY_RESPONSE
HALLMARK_PANCREAS_BETA_CELLS
HALLMARK_TNFA_SIGNALING_VIA_NFKB
Ranked List Metric
10
5
0
−5
−10
5000
10000
15000
Rank in Ordered Dataset

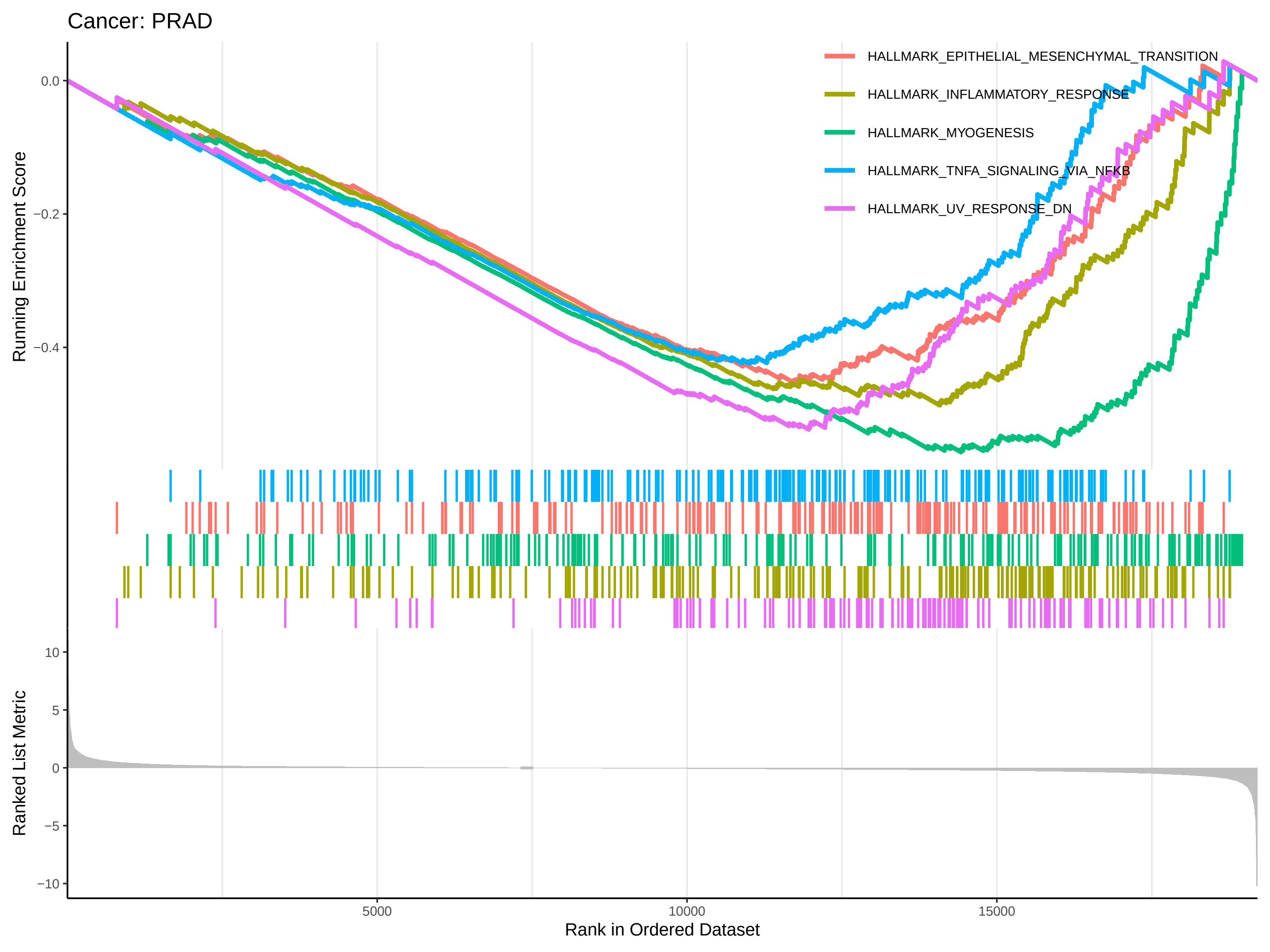

Cancer: PRAD
Running Enrichment Score
0.0
−0.2
−0.4
HALLMARK_EPITHELIAL_MESENCHYMAL_TRANSITION
HALLMARK_INFLAMMATORY_RESPONSE
HALLMARK_MYOGENESIS
HALLMARK_TNFA_SIGNALING_VIA_NFKB
HALLMARK_UV_RESPONSE_DN
Ranked List Metric
10
5
0
−5
−10
5000
10000
15000
Rank in Ordered Dataset

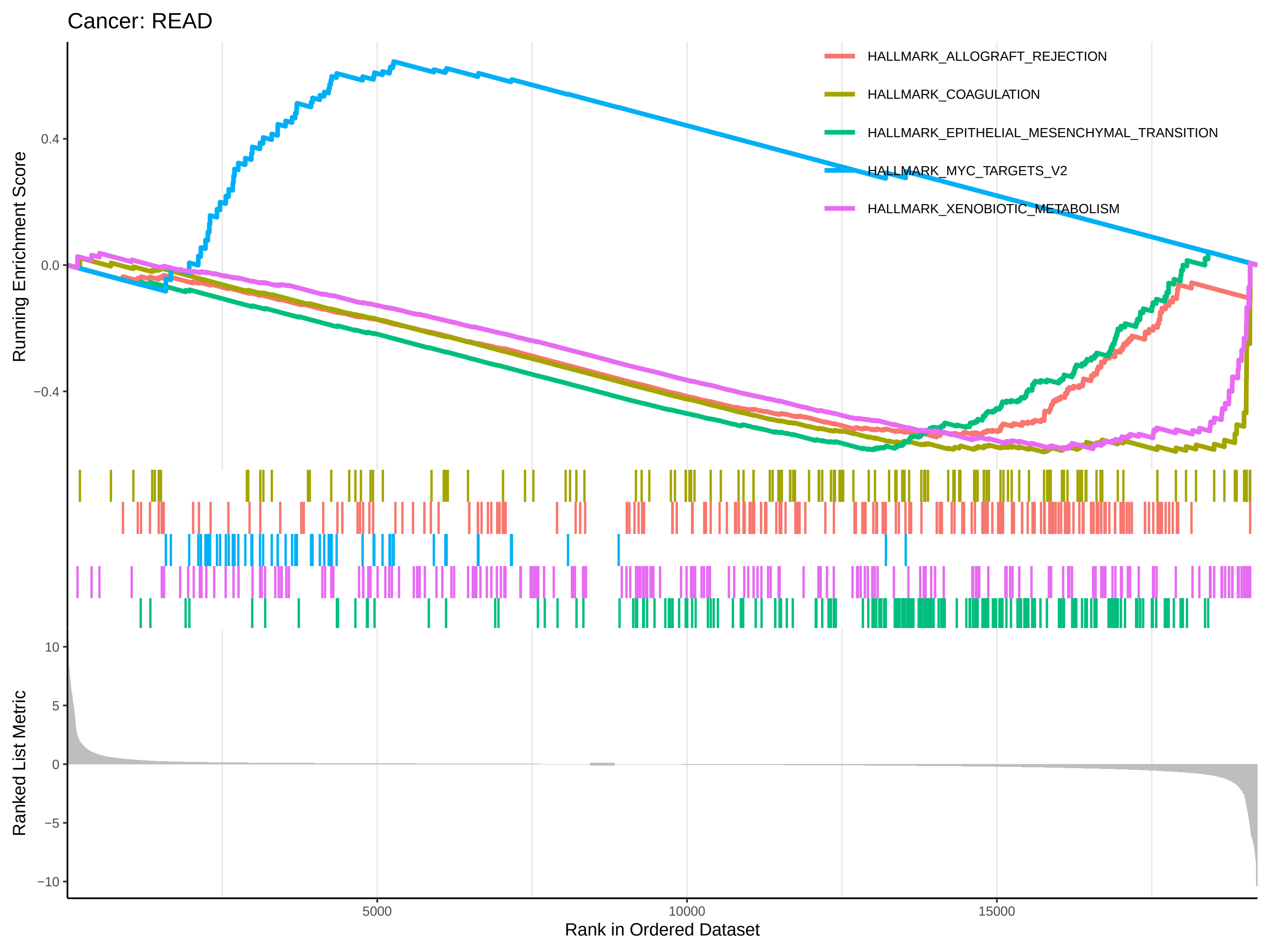
Cancer: READ
HALLMARK_ALLOGRAFT_REJECTION
HALLMARK_COAGULATION
HALLMARK_EPITHELIAL_MESENCHYMAL_TRANSITION
HALLMARK_MYC_TARGETS_V2
HALLMARK_XENOBIOTIC_METABOLISM
Running Enrichment Score
0.4
0.0
−0.4
Ranked List Metric
10
5
0
−5
−10
5000
10000
15000
Rank in Ordered Dataset

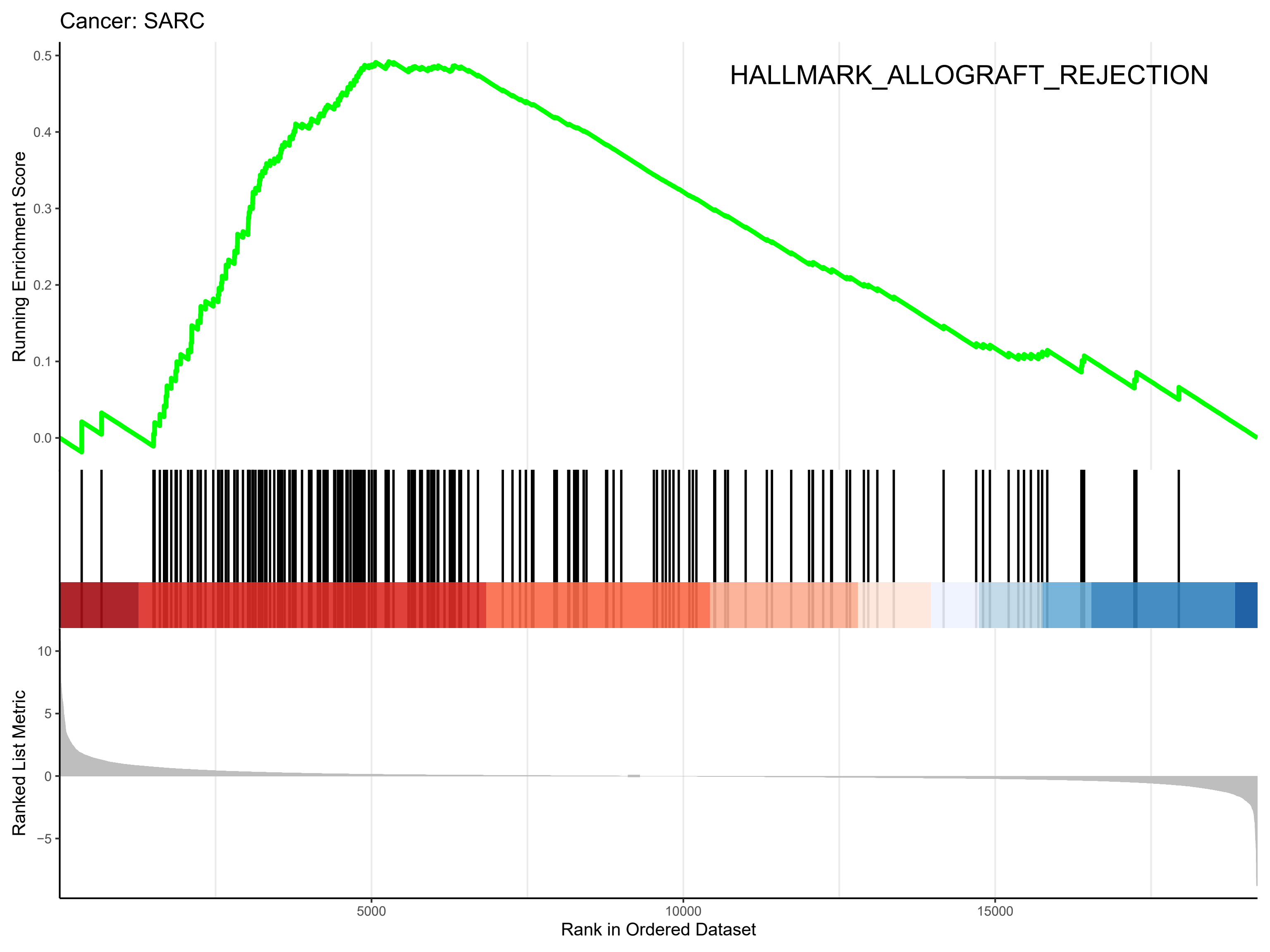
Cancer: SARC
HALLMARK_ALLOGRAFT_REJECTION
Running Enrichment Score
0.5
0.4
0.3
0.2
0.1
0.0
Ranked List Metric
10
5
0
−5
5000
10000
15000
Rank in Ordered Dataset

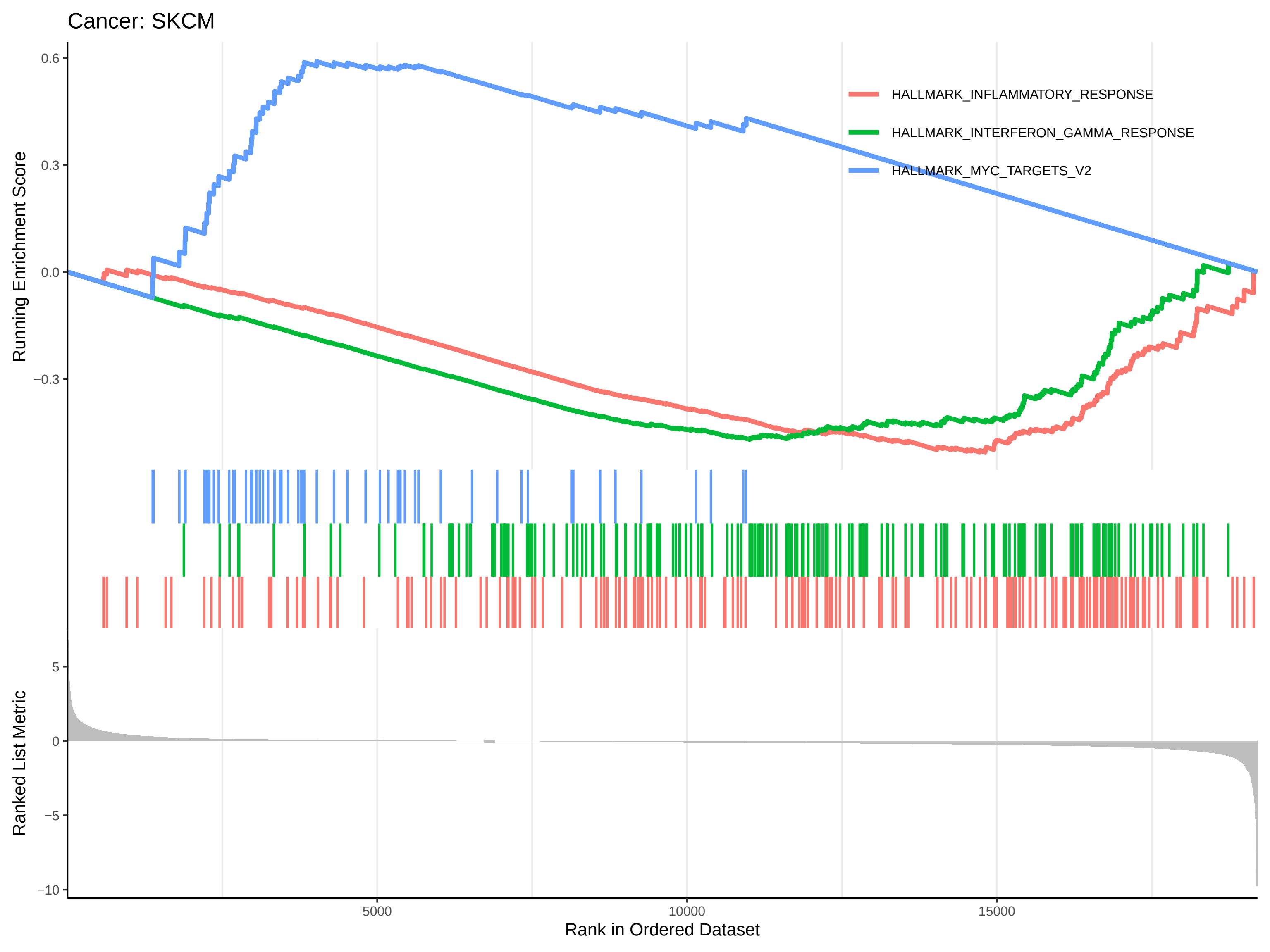

Cancer: SKCM
HALLMARK_INFLAMMATORY_RESPONSE
HALLMARK_INTERFERON_GAMMA_RESPONSE
HALLMARK_MYC_TARGETS_V2
Running Enrichment Score
0.6
0.3
0.0
−0.3
Ranked List Metric
5
0
−5
−10
5000
10000
15000
Rank in Ordered Dataset

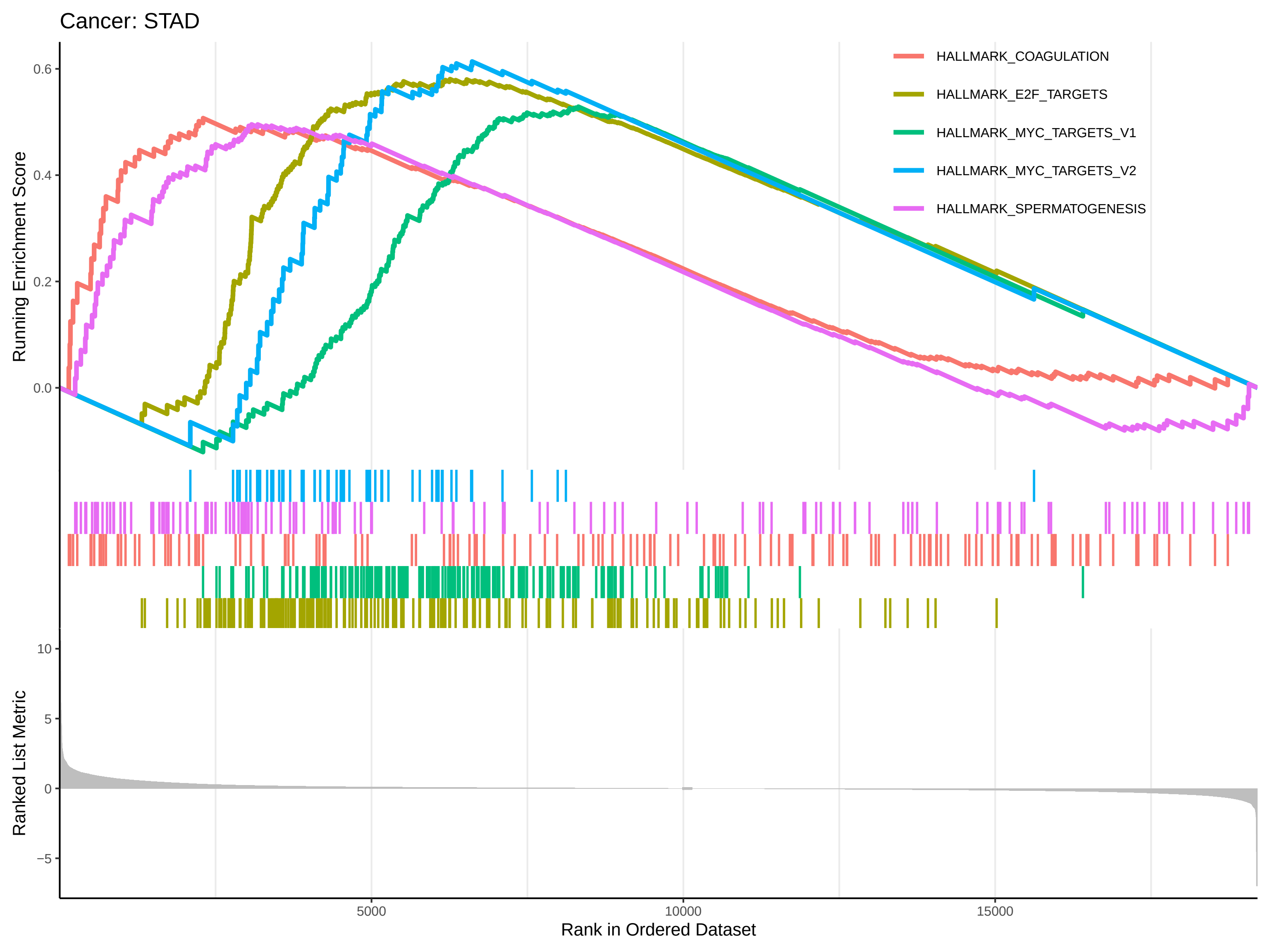

Cancer: STAD
HALLMARK_COAGULATION
HALLMARK_E2F_TARGETS
HALLMARK_MYC_TARGETS_V1
HALLMARK_MYC_TARGETS_V2
HALLMARK_SPERMATOGENESIS
Running Enrichment Score
0.6
0.4
0.2
0.0
Ranked List Metric
10
5
0
−5
5000
10000
15000
Rank in Ordered Dataset

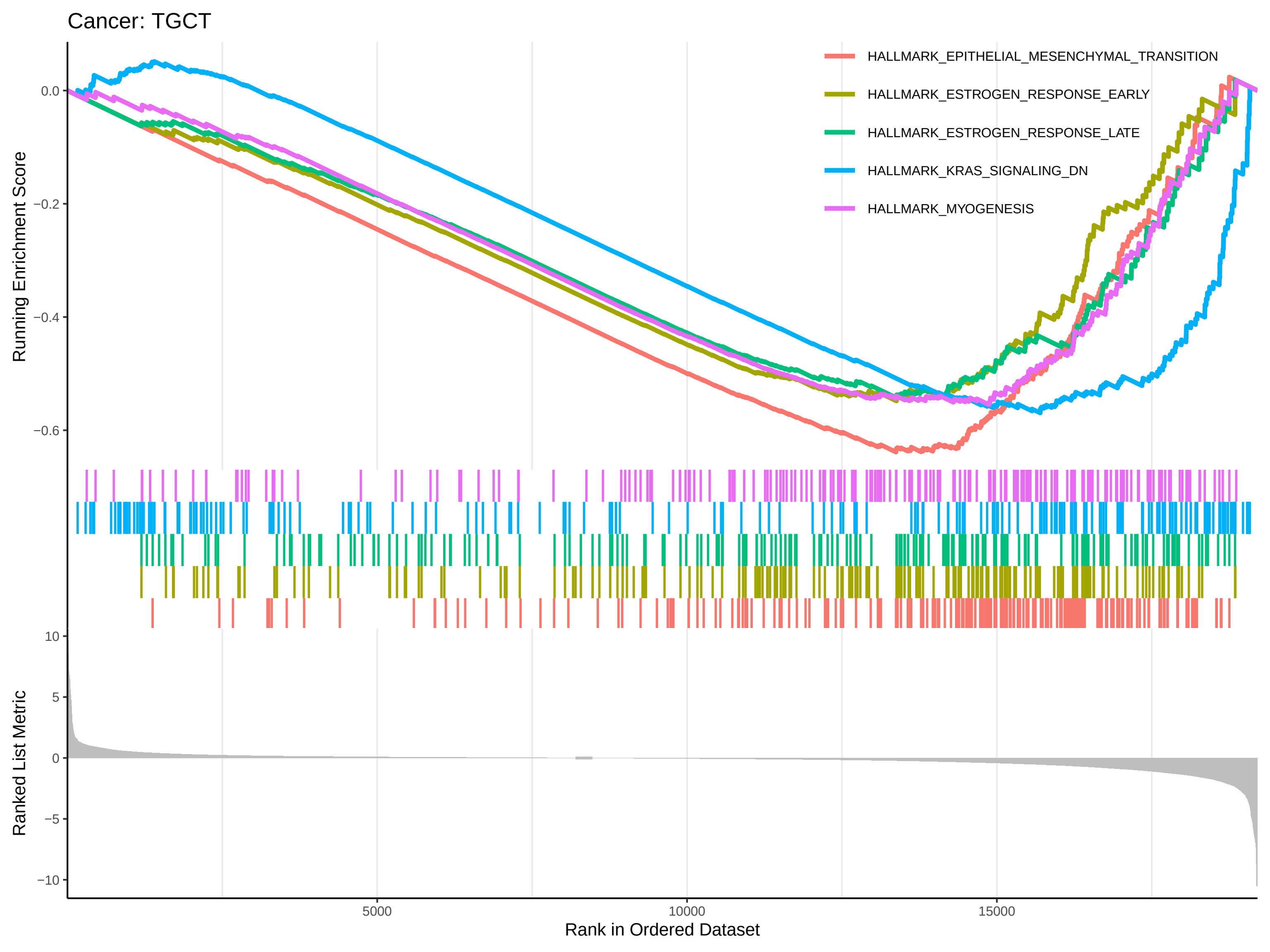
Cancer: TGCT
HALLMARK_EPITHELIAL_MESENCHYMAL_TRANSITION
HALLMARK_ESTROGEN_RESPONSE_EARLY
HALLMARK_ESTROGEN_RESPONSE_LATE
HALLMARK_KRAS_SIGNALING_DN
HALLMARK_MYOGENESIS
Running Enrichment Score
0.0
−0.2
−0.4
−0.6
Ranked List Metric
10
5
0
−5
−10
5000
10000
15000
Rank in Ordered Dataset

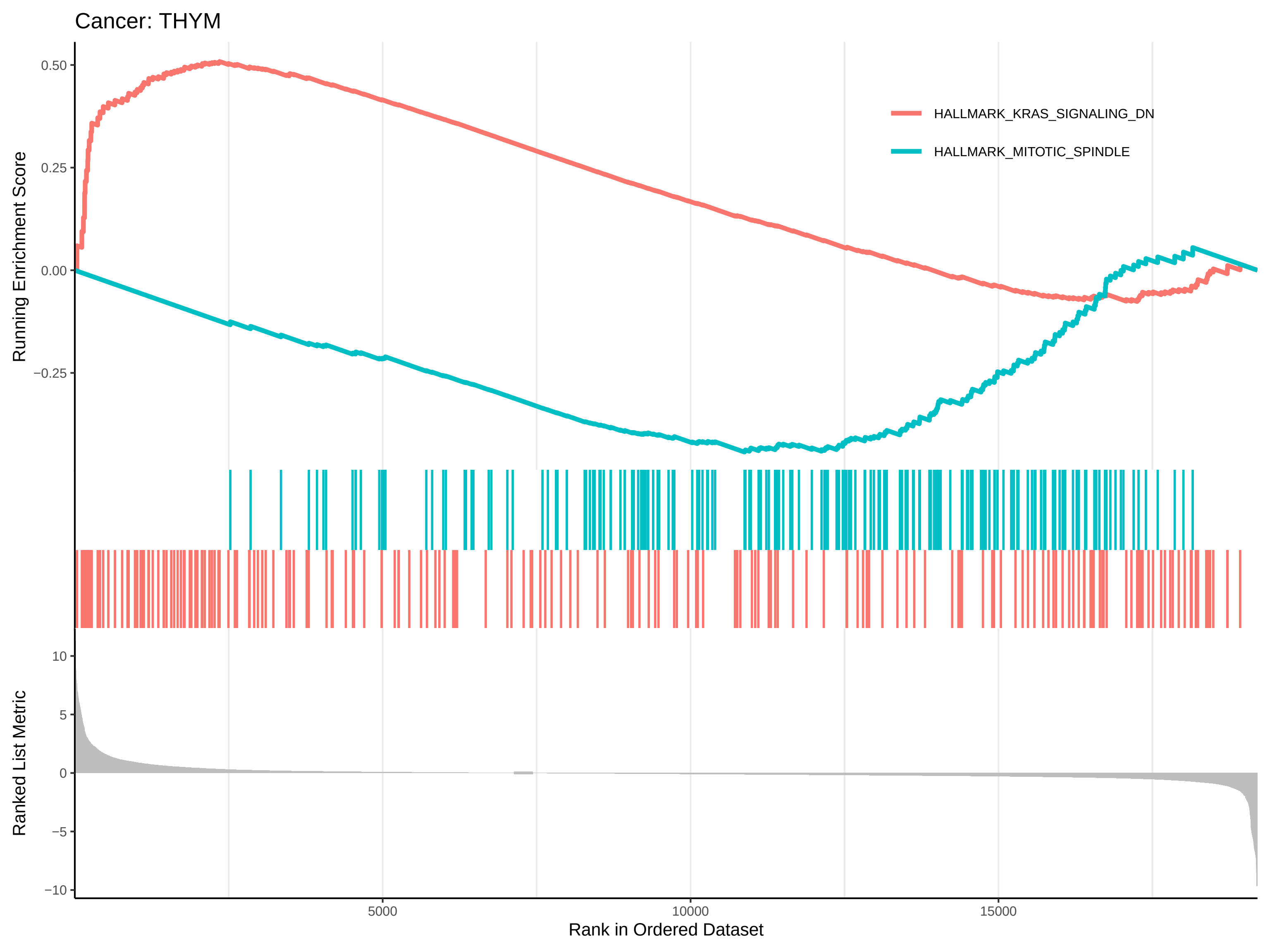
Cancer: THYM
Running Enrichment Score
0.50
0.25
0.00
−0.25
HALLMARK_KRAS_SIGNALING_DN
HALLMARK_MITOTIC_SPINDLE
Ranked List Metric
10
5
0
−5
−10
5000
10000
15000
Rank in Ordered Dataset

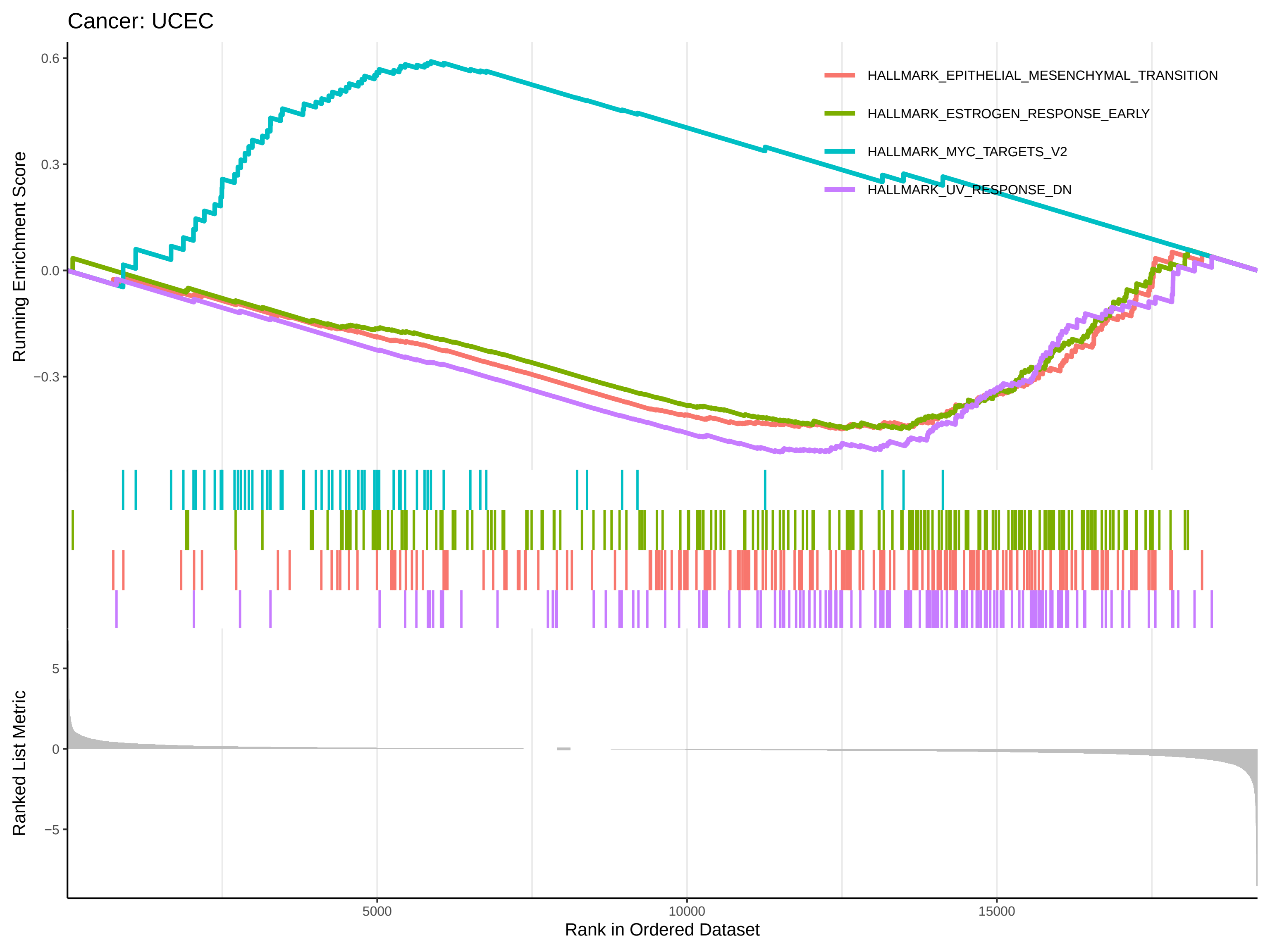

Cancer: UCEC
HALLMARK_EPITHELIAL_MESENCHYMAL_TRANSITION
HALLMARK_ESTROGEN_RESPONSE_EARLY
HALLMARK_MYC_TARGETS_V2
HALLMARK_UV_RESPONSE_DN
Running Enrichment Score
0.6
0.3
0.0
−0.3
Ranked List Metric
5
0
−5
5000
10000
15000
Rank in Ordered Dataset

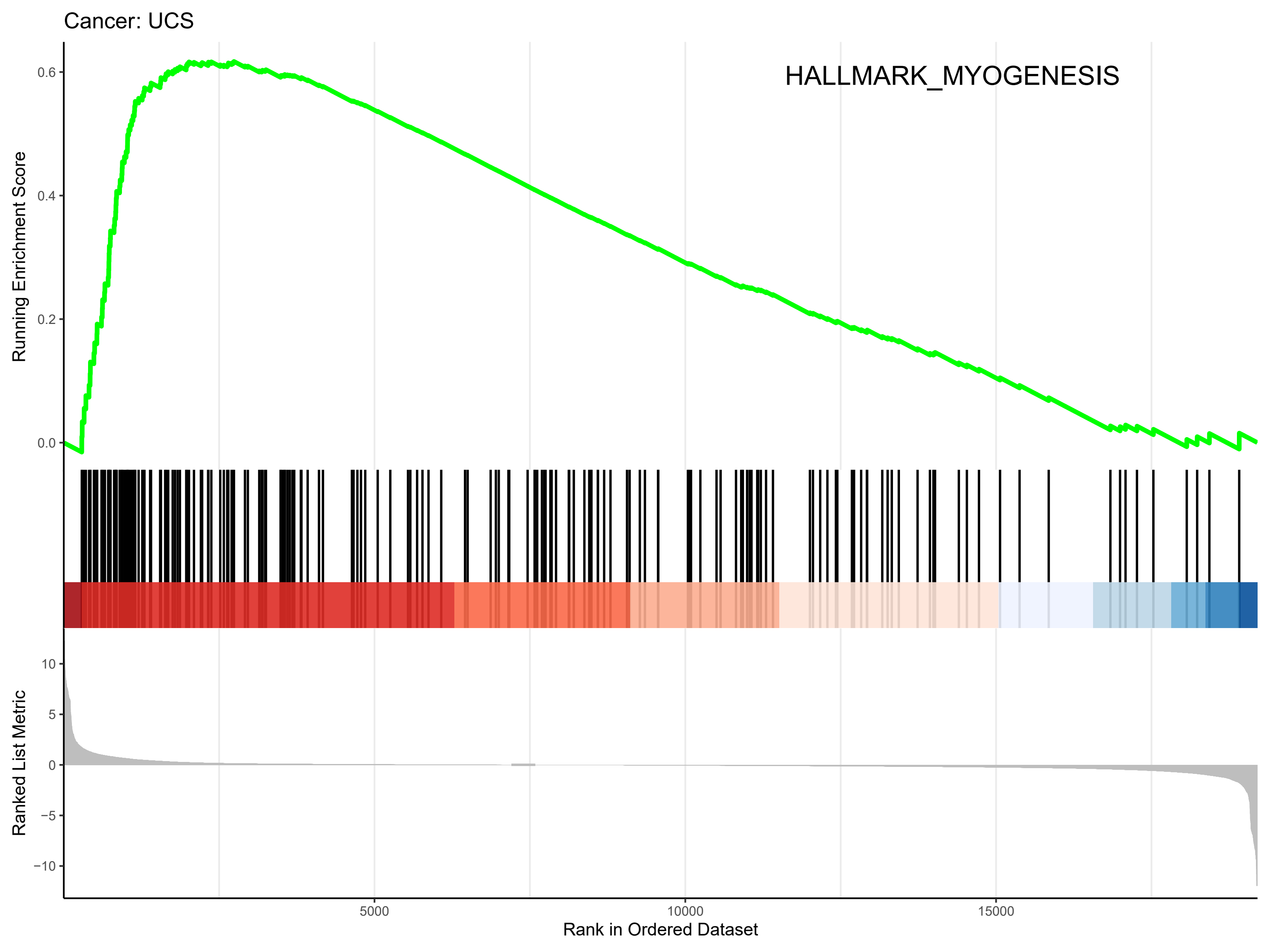

Cancer: UCS
HALLMARK_MYOGENESIS
Running Enrichment Score
0.6
0.4
0.2
0.0
Ranked List Metric
10
5
0
−5
−10
5000
10000
15000
Rank in Ordered Dataset

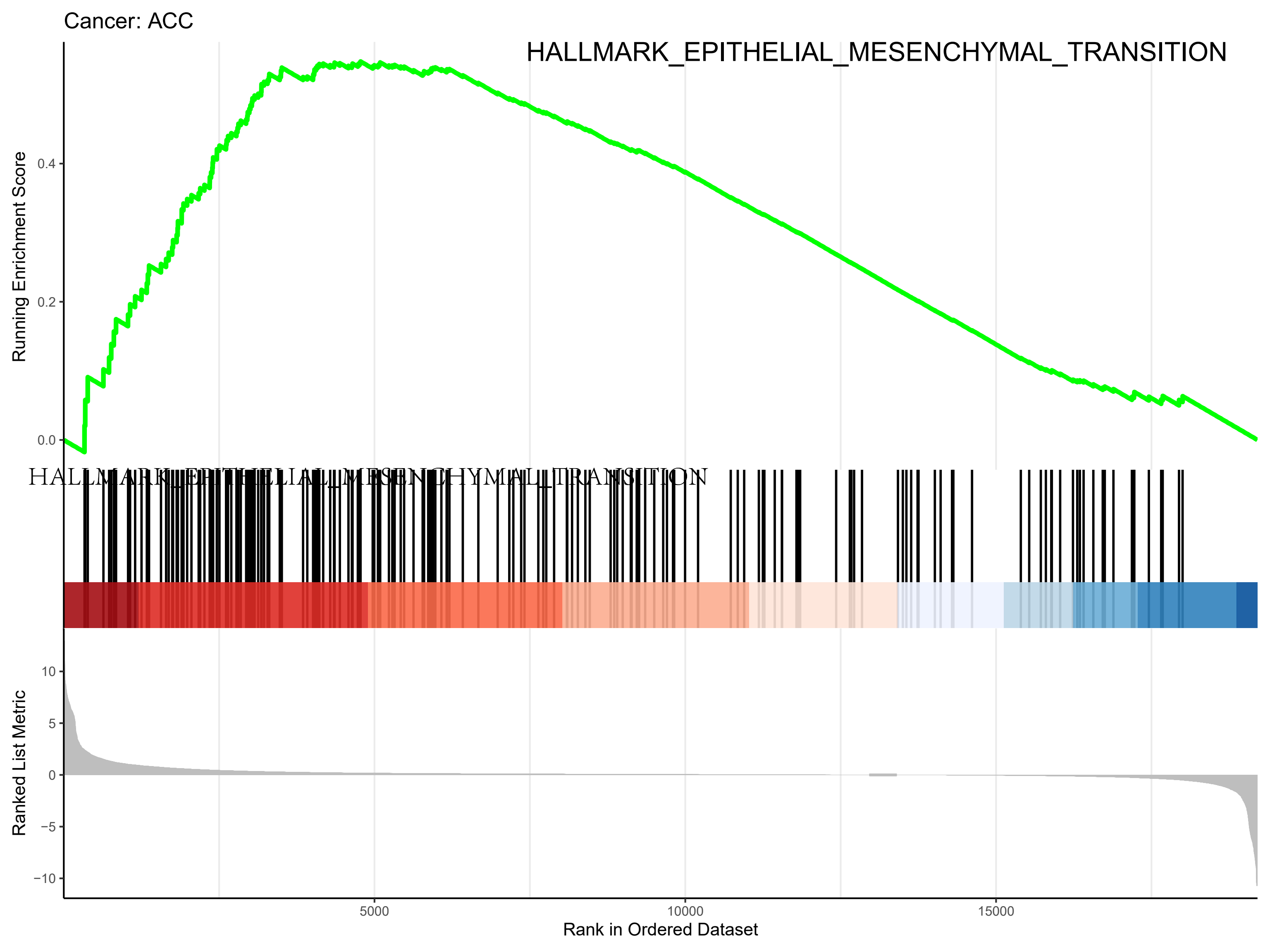

Cancer: ACC
HALLMARK_EPITHELIAL_MESENCHYMAL_TRANSITION
Running Enrichment Score
0.4
0.2
0.0
HALLMARK_EPITHELIAL_MESENCHYMAL_TRANSITION
Ranked List Metric
10
5
0
−5
−10
5000
10000
15000
Rank in Ordered Dataset

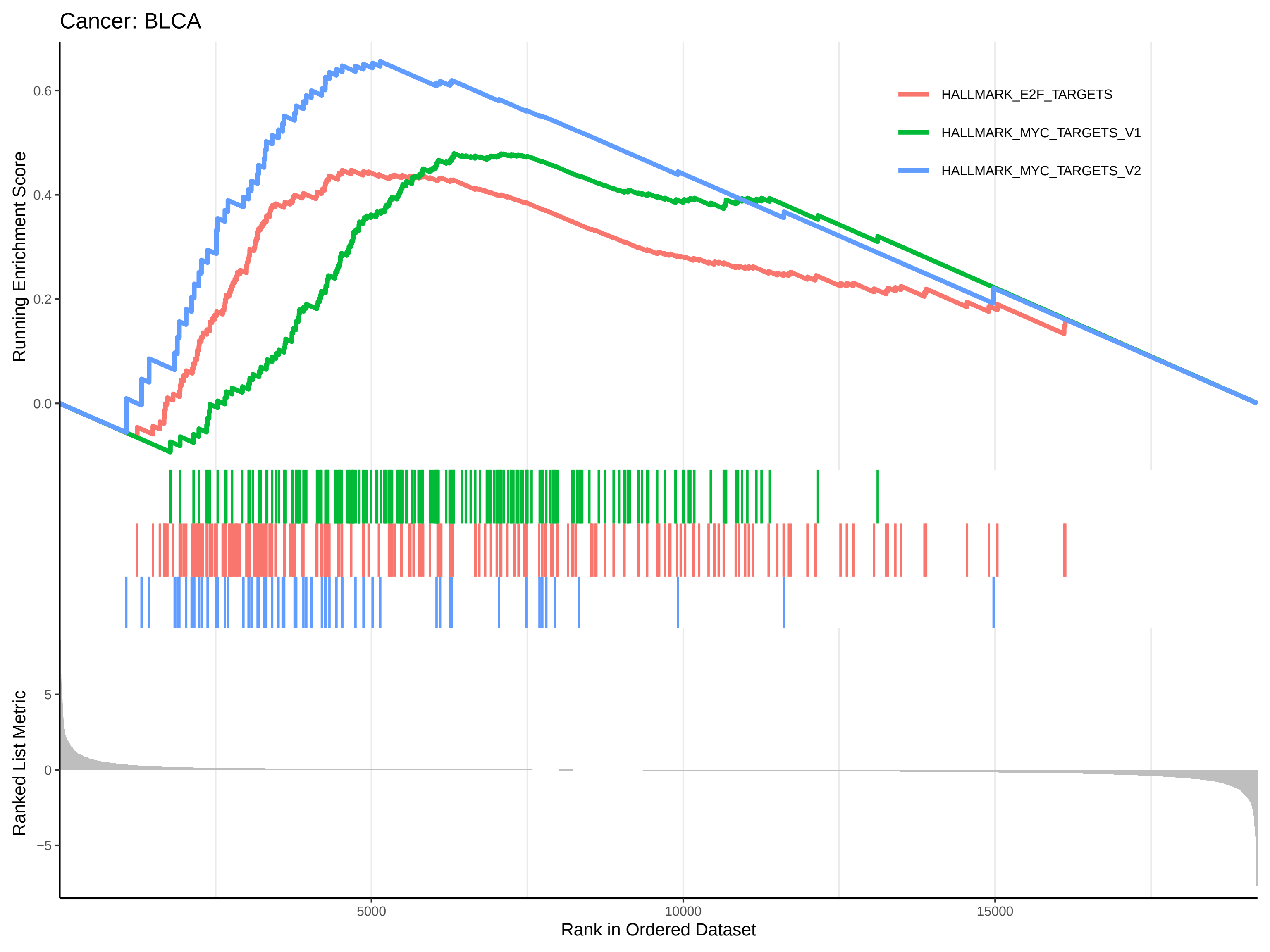

Cancer: BLCA
Running Enrichment Score
0.6
0.4
0.2
0.0
HALLMARK_E2F_TARGETS
HALLMARK_MYC_TARGETS_V1
HALLMARK_MYC_TARGETS_V2
Ranked List Metric
5
0
−5
5000
10000
15000
Rank in Ordered Dataset

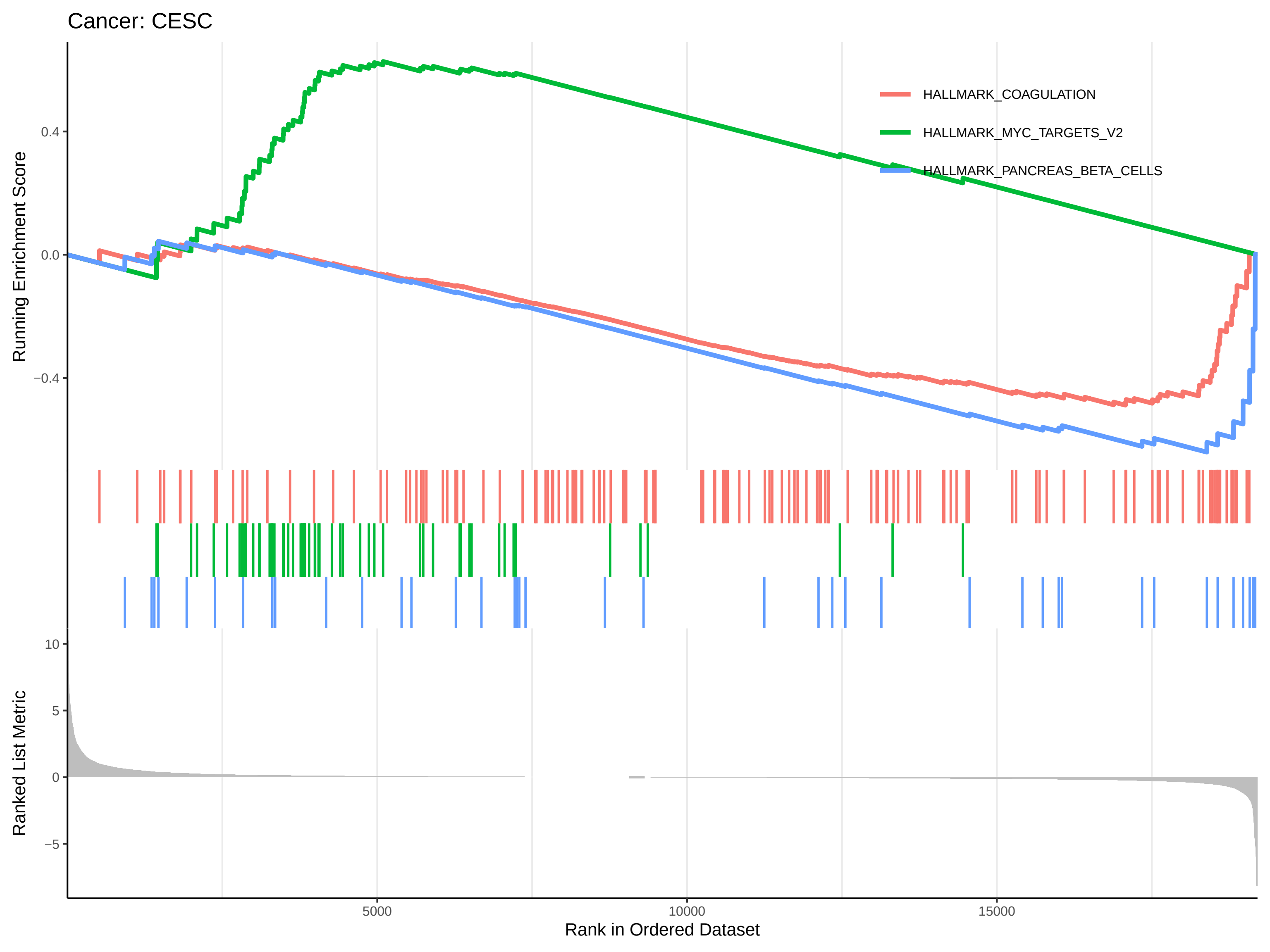

Cancer: CESC
HALLMARK_COAGULATION
HALLMARK_MYC_TARGETS_V2
HALLMARK_PANCREAS_BETA_CELLS
Running Enrichment Score
0.4
0.0
−0.4
Ranked List Metric
10
5
0
−5
5000
10000
15000
Rank in Ordered Dataset

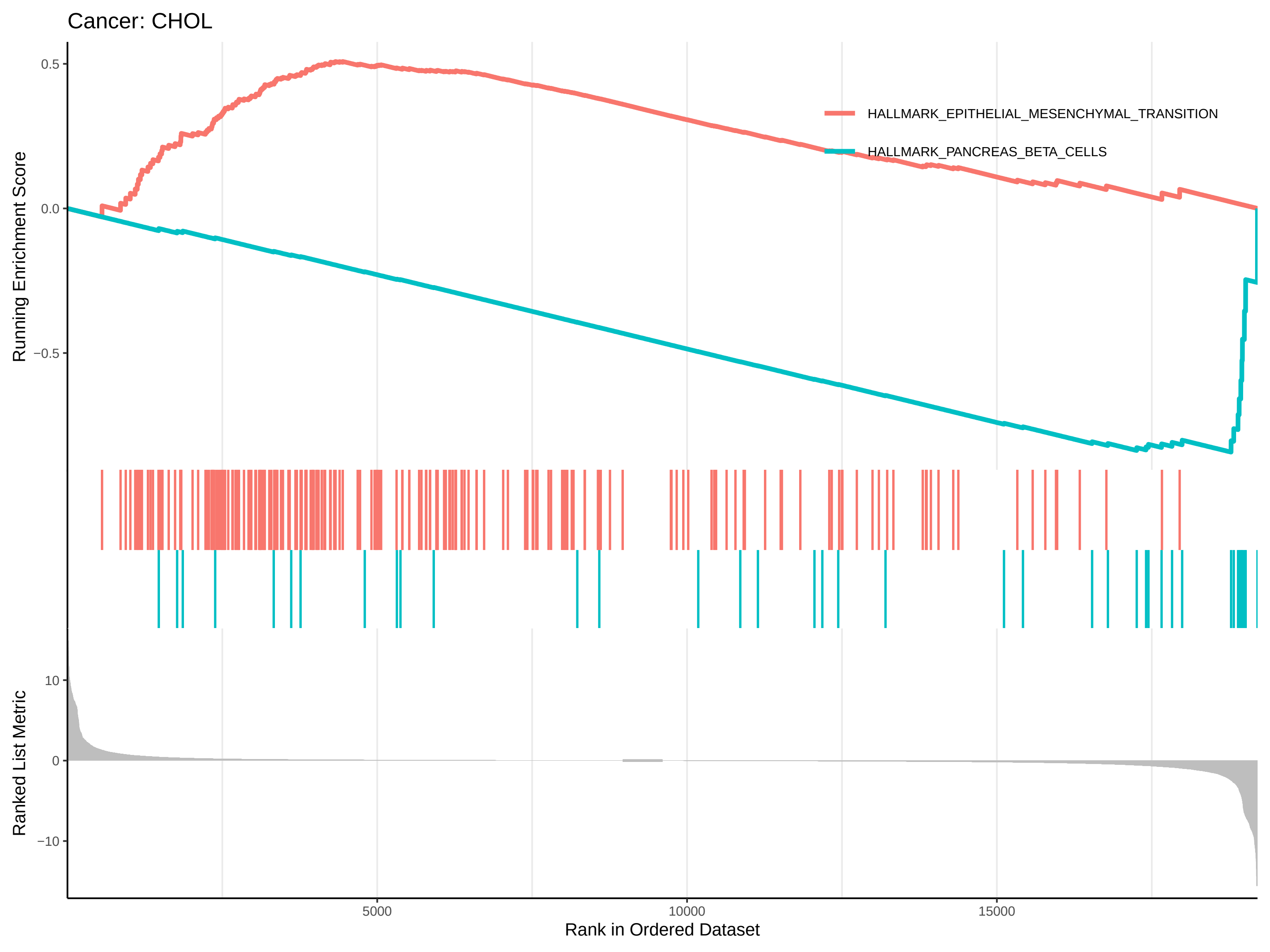

Cancer: CHOL
Running Enrichment Score
0.5
0.0
−0.5
HALLMARK_EPITHELIAL_MESENCHYMAL_TRANSITION
HALLMARK_PANCREAS_BETA_CELLS
Ranked List Metric
10
0
−10
5000
10000
15000
Rank in Ordered Dataset

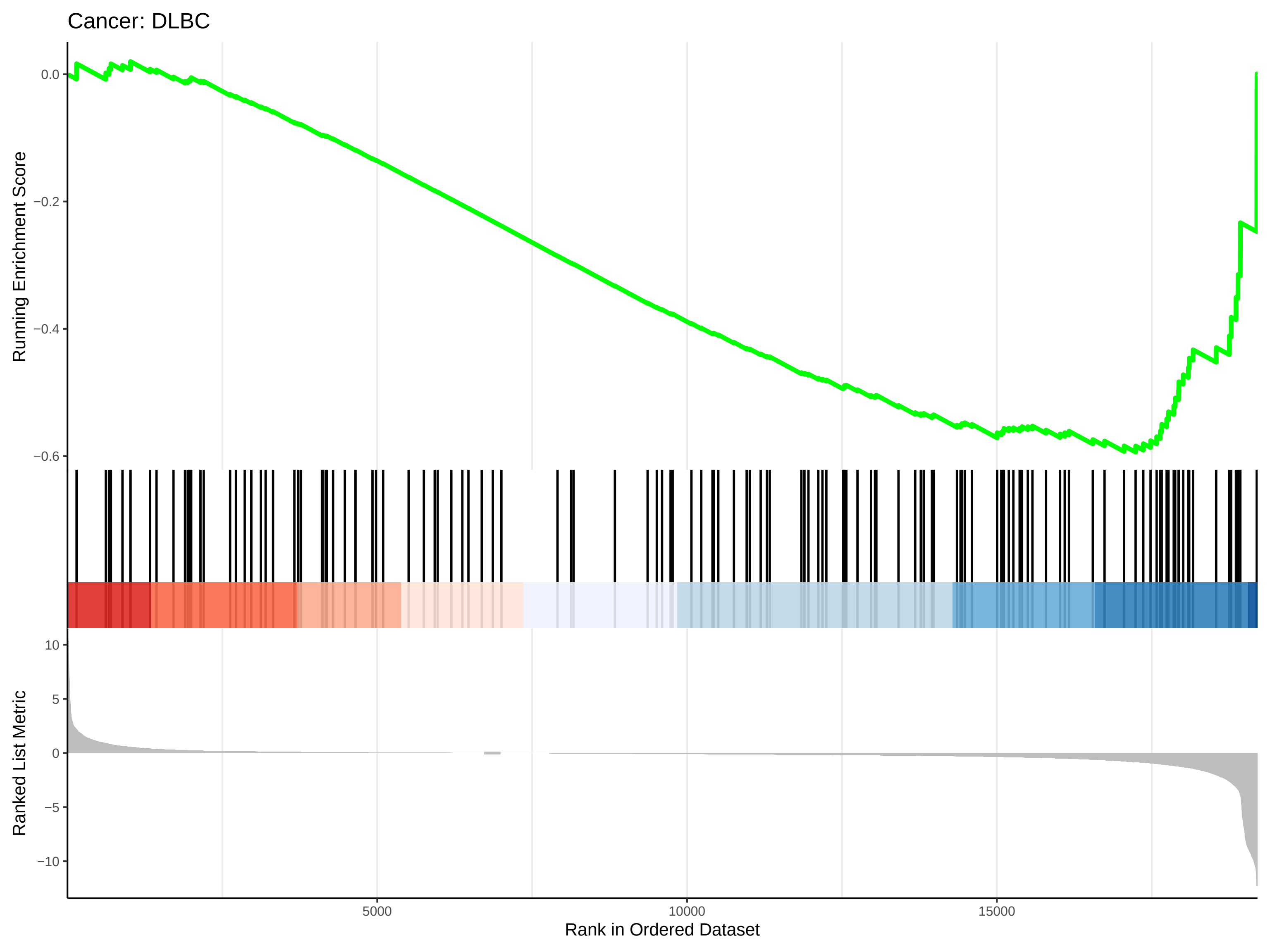

Cancer: DLBC
Running Enrichment Score
0.0
−0.2
−0.4
−0.6
Ranked List Metric
10
5
0
−5
−10
5000
10000
15000
Rank in Ordered Dataset

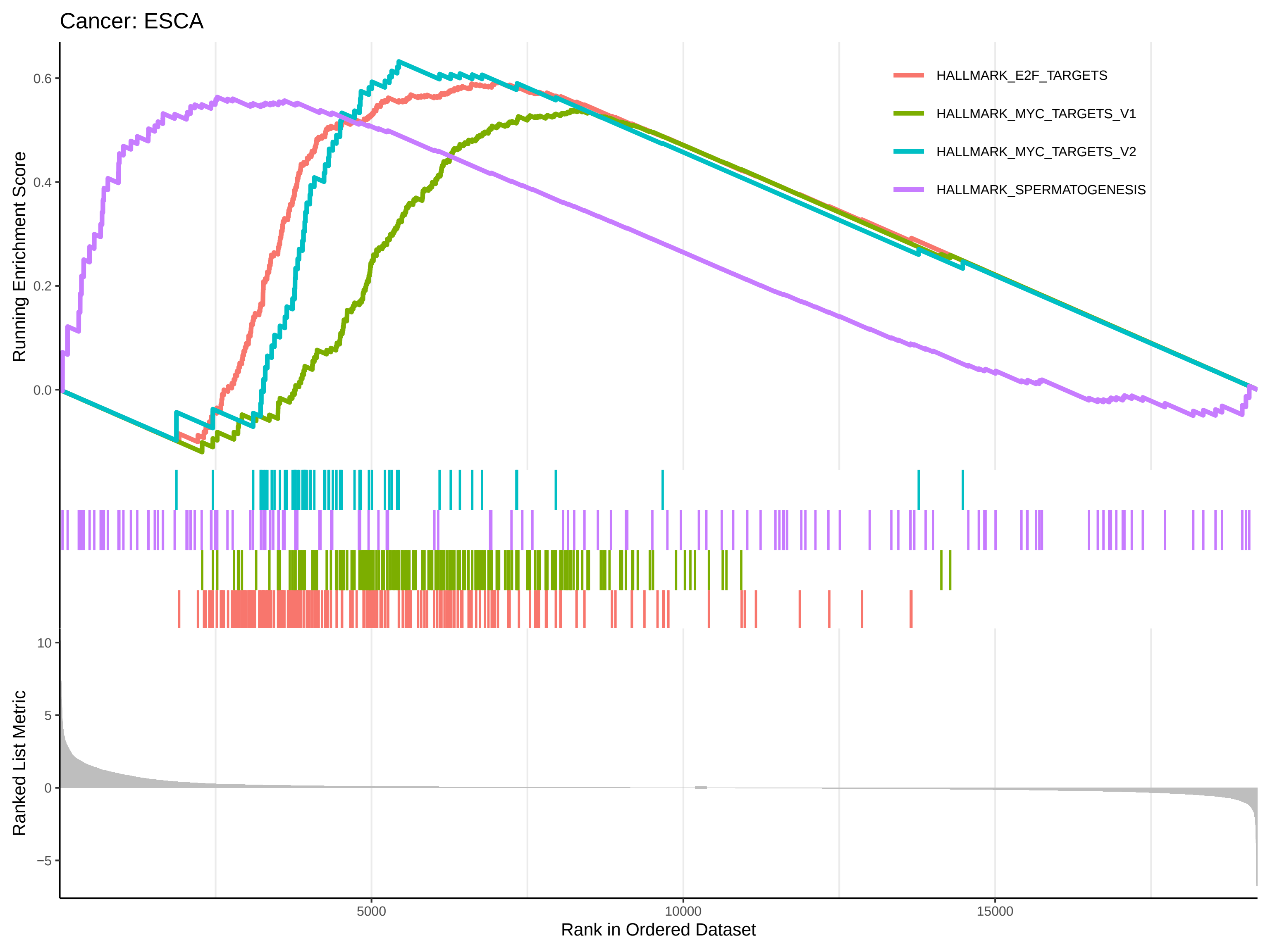

Cancer: ESCA
Running Enrichment Score
0.6
0.4
0.2
0.0
HALLMARK_E2F_TARGETS
HALLMARK_MYC_TARGETS_V1
HALLMARK_MYC_TARGETS_V2
HALLMARK_SPERMATOGENESIS
Ranked List Metric
10
5
0
−5
5000
10000
15000
Rank in Ordered Dataset

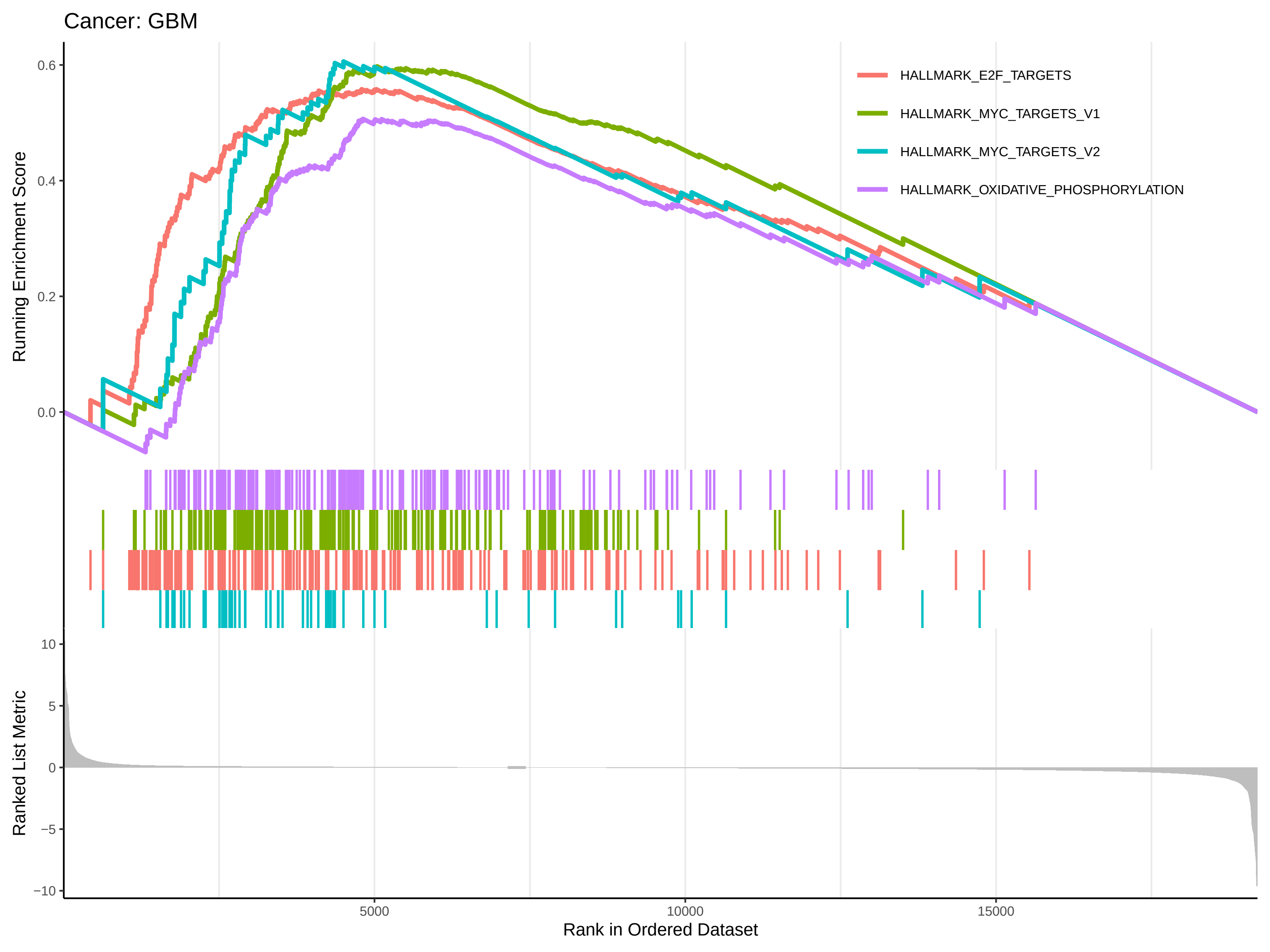
Cancer: GBM
Running Enrichment Score
0.6
0.4
0.2
0.0
HALLMARK_E2F_TARGETS
HALLMARK_MYC_TARGETS_V1
HALLMARK_MYC_TARGETS_V2
HALLMARK_OXIDATIVE_PHOSPHORYLATION
Ranked List Metric
10
5
0
−5
−10
5000
10000
15000
Rank in Ordered Dataset
